# Fucoidan-coated CuS nanoparticles for chemo-and photothermal therapy against cancer

**DOI:** 10.18632/oncotarget.23898

**Published:** 2018-01-03

**Authors:** Bian Jang, Madhappan Santha Moorthy, Panchanathan Manivasagan, Li Xu, Kyeongeun Song, Kang Dae Lee, Minseok Kwak, Junghwan Oh, Jun-O Jin

**Affiliations:** ^1^ Shanghai Public Health Clinical Center, Shanghai Medical College, Fudan University, Jinshan District, Shanghai, China; ^2^ Marine-Integrated Bionics Research Center, Pukyong National University, Busan, South Korea; ^3^ Department of Biomedical Engineering and Center for Marine-Integrated Biomedical Technology (BK21 Plus), Busan, South Korea; ^4^ Interdisciplinary Program of Biomedical Mechanical and Electrical Engineering, Busan, South Korea; ^5^ Department of Chemistry, Pukyong National University, Busan, South Korea; ^6^ Department of Otolaryngology-Head and Neck Surgery, Kosin University College of Medicine, Busan, Korea

**Keywords:** fucodian, copper sulfide nanoparticles, photothermal therapy, chemotherapy, apoptosis

## Abstract

In advanced cancer therapy, the combinational therapeutic effect of photothermal therapy (PTT) using near-infrared (NIR) light-responsive nanoparticles (NPs) and anti-cancer drug delivery-mediated chemotherapy has been widely applied. In the present study, using a facile, low-cost, and solution-based method, we developed and synthesized fucoidan, a natural polymer isolated from seaweed that has demonstrated anti-cancer effect, and coated NPs with it as an ideal candidate in chemo-photothermal therapy against cancer cells. Fucoidan-coated copper sulfide nanoparticles (F-CuS) act not only as a nanocarrier to enhance the intracellular delivery of fucoidan but also as a photothermal agent to effectively ablate different cancer cells (e.g., HeLa, A549, and K562), both *in vitro* and *in vivo*, with the induction of apoptosis under 808 nm diode laser irradiation. These results point to the potential usage of F-CuS in treating human cancer.

## INTRODUCTION

For the delivery of anti-cancer drugs, nanoparticles (NPs) have been used as drug carriers; they target tumor cells through the phagocytosis or endocytosis of NPs, and therefore, they are suitable for the delivery of chemotherapeutics in cancer treatment [1]. Since the natural products show an efficient anti-cancer effect without imposing any toxicity on normal cells, they are promising as an alternative to chemical drugs [2]. Fucoidan is a sulfated polysaccharide extracted from marine brown seaweeds; it possesses various biological properties such as anti-coagulant, anti-virus, anti-cancer, and immune modulation [3–7]. In particular, the anti-cancer effect of fucoidan against various types of cancer—including breast cancer, lung cancer, cervical cancer, and leukemia—has already been well defined [6, 8–10]. Although fucoidan-mediated anti-cancer effect by nanomaterials have been used in cancer treatment [11–13], combination of hyperthermia and anti-cancer effects of fucoidan has not yet been studied.

Photothermal therapy (PTT), an emerging alternative or supplement to conventional cancer treatments, has great advantages, such as minimal invasion, cancer-site specificities, and fast recovery [14]. PTT employs a near-infrared (NIR) light source and a photoabsorber that absorbs and converts light energy into heat to ablate cancer cells. As the interest in PTT grows, various NIR-responsive nanoparticles have been developed, such as gold nanorods [15], carbon nanotubes [16], magnetic iron oxide nanoparticles [17], and copper sulfide nanoparticles [18]; some NPs having proven therapeutic effects and biocompatibility are under clinical trial [19]. Copper sulfide nanoparticles show great biocompatibility, photothermal conversion efficiency, and photostability within the size of 3–30 nm, and they are cost effective when used in synthesis; they satisfy the requirements as a promising candidate among photothermal agents [20].

The apoptosis, so-called programmed cell death, plays a key role in successful cancer treatment. Apoptosis has mainly two Apoptosis has mainly two pathways: mitocontria-dependent and independent. Unlike necrosis, apoptosis develops apoptotic bodies that can be phagocytosed by antigen- presenting cells, such as dendritic cells and macrophages, and consequently promotes cancer antigen-specific immune activation and prevent tumor growth [21, 22]. Recently, to improve the therapeutic effect against cancer, a combination of chemotherapy and PTT has been widely studied. Although it has attracted great interest for its efficient delivery of anti-cancer drugs into cancer cells by NPs and additional therapeutic effects by PTT [23–25], there is no biological benefit, the chemotherapy reagents such as doxorubicin and paclitaxel induces necrotic cancer cell death [26, 27]. Therefore, the induction of apoptosis through treatments that combine chemotherapy and PTT against cancer cells needs to be carefully investigated.

In this study, we introduce a facile electrostatic method to synthesize small and highly biocompatible fucoidan-coated copper sulfide nanoparticles (F-CuS) for chemo-photothermal therapy. We hypothesized that F-CuS may promote cancer cell apoptosis through the improved intracellular delivery of fucoidan and the photothermal effect of NIR irradiation; therefore, the current study was undertaken to test this hypothesis against HeLa, A549, and K562 cells.

## RESULTS AND DISCUSSION

### Preparation and characterization of F-CuS NPs

**Figure 1:**
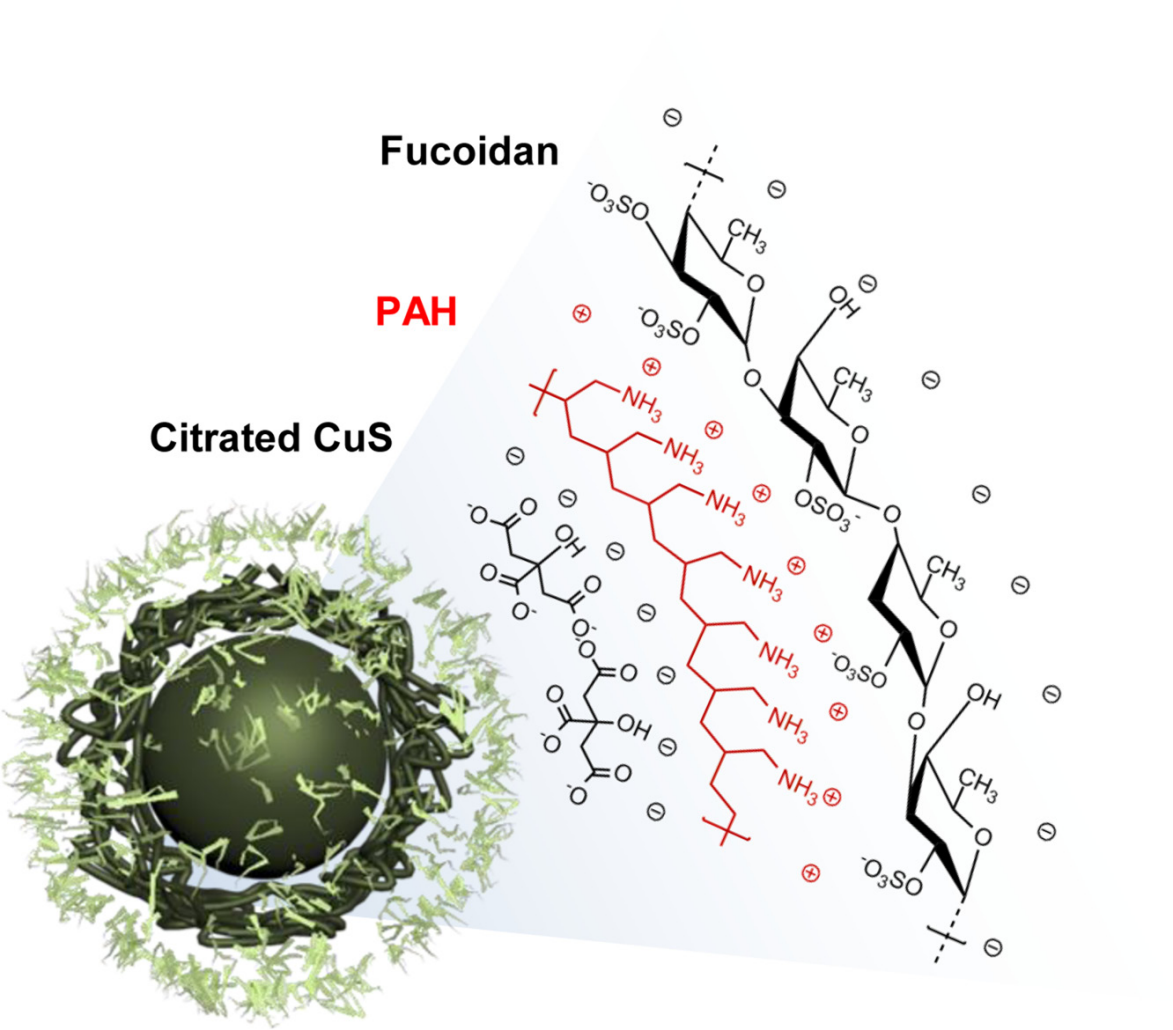
Structural illustration of F-CuS prepared for chemo- and photothermal cancer therapy

**Figure 2:**
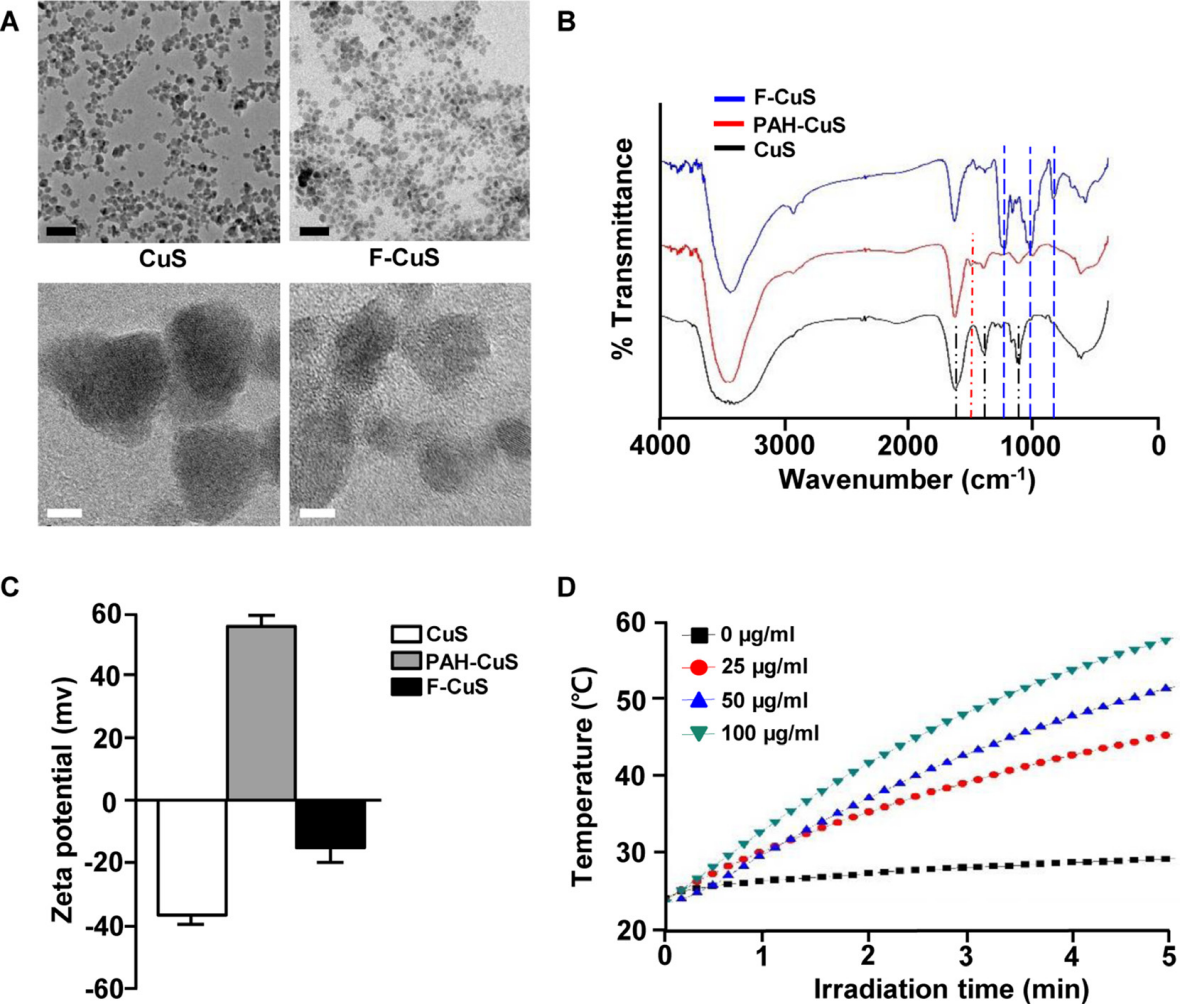
Characterization of F-CuS (**A**) FE-TEM images of CuS and F-CuS. Scale bars: 50 nm (black) and 5 nm (white). (**B**) FT-IR spectra of CuS (black), PAH-CuS (red), and F-CuS (blue). (**C**) Zeta potential value of each NP. (**D**) Photothermal heating curves of different concentrations of F-CuS dispersed in water, irradiated for 5 min with an 808 nm laser at a power density of 2 W/cm^2^.

We first synthesized sodium citrate-stabilized copper sulfide nanoparticles (CuS) and coated the NPs using the layer-by-layer (LbL) technique and alternating poly-cationic and anionic substances [i.e., polyallylamine hydrochloride (PAH) and fucoidan from *Fucus vesicuosus*, respectively (Figure 1)] [28, 29]. The intermediate adhesive layer, the PAH coating, not only improves the stability of NPs [30] but also acts as a linker by introducing the positively charged functional group onto the negatively charged CuS surface [29]. Then, the surface of PAH-CuS was coated by fucoidan by creating a strong electrostatic interaction between PAH (NH_3_^+^) and fucoidan (SO_3_^–^). Additionally, we hypothesized that the LbL-fabricated NPs could be pH-responsive within cancerous cells [31, 32]. On transmission electron microscopy images, we observed spherical CuS and F-CuS with an average size of 10 nm (Figures 2A and Supplementary Figure 1). X-ray diffraction and energy-dispersive x-ray spectroscopy (EDS) were used to determine and confirm the crystallization and purity of the synthesized CuS, relative to the standard data from the Joint Committee on Powder Diffraction Standards card (06-0464). The strong and sharp peaks prove the fair crystallinity of the synthesized NP, while the lack of other peaks testifies the high purity of the substance (Supplementary Figure 2). Successful coatings of PAH and fucoidan onto CuS were confirmed by performing Fourier-transform infrared spectroscopy (FT-IR) and examining zeta potential. As shown in Figure 2B, all three FT-IR spectra showed the Cu-S stretching at 614 cm^–1^. Since the citrate worked as a stabilizer in CuS, peaks indicating C–O (1114 cm^–1^), O–H (1389 cm^–1^), and C = O (1626 cm^–1^) were present in the CuS spectra. After applying the PAH coating onto the CuS, a new peak appeared at 1498 cm^–1^, indicating the bending mode of amine (N–H). After applying the fucoidan coating onto PAH-CuS, the formation of new peaks of S = O (840, 1230 cm^–1^) and a saccharide ring (1024 cm^–1^) were detected, which indicate the presence of fucoidan. In addition, zeta potential values changed from –37.40 (± 0.99) mV (CuS) to +56.35 (± 2.28) mV (PAH-CuS) after coating of PAH and then to –16.08 (± 1.84) mV (F-CuS) after coating of fucoidan (Figure 2C). Thus, these data indicate that CuS had been successfully coated with PAH and fucoidan.

### Photothermal property of F-CuS

A remarkable feature of CuS is the broad and strong absorption in the NIR region (700–1100 nm) for utilizing the heat generated by the vibrational energy of the d–d transition of Cu^2+^ (Supplementary Figure 3A). To evaluate the photothermal effect, the temperature of aqueous F-CuS samples at different concentrations were measured under laser irradiation (2 W/cm^2^) at 808 nm for 5 min. As anticipated, higher concentrations lead to higher temperature increases under NIR irradiation, where 50 and 100 μg/mL F-CuS solutions reached 50°C within 4 and 3 min, respectively; the phosphate buffered saline (PBS)-mediated temperature increase, in comparison, was only 5°C in 5 min (Figure 2D). Moreover, the great photostability of F-CuS was confirmed by measuring its UV-vis absorption spectra before and after the laser irradiation, and the stable photothermal heating efficiency of NPs during 5 cycles of laser ON/OFF experiment was performed to prove the great photostability of F-CuS (Supplementary Figure 3C and 3D). Therefore, we concluded that the F-CuS system is a good candidate for PTT material, given its proven stability under experimental conditions. In addition, to check the stability of the sample, the photographs and the UV-vis absorption of F-CuS dispersed in cell media before and after 2 h were measured and we observed no big differences in both data (Supplementary Figure 3E).

### F-CuS mediated anti-cancer effect *in vitro*

**Figure 3:**
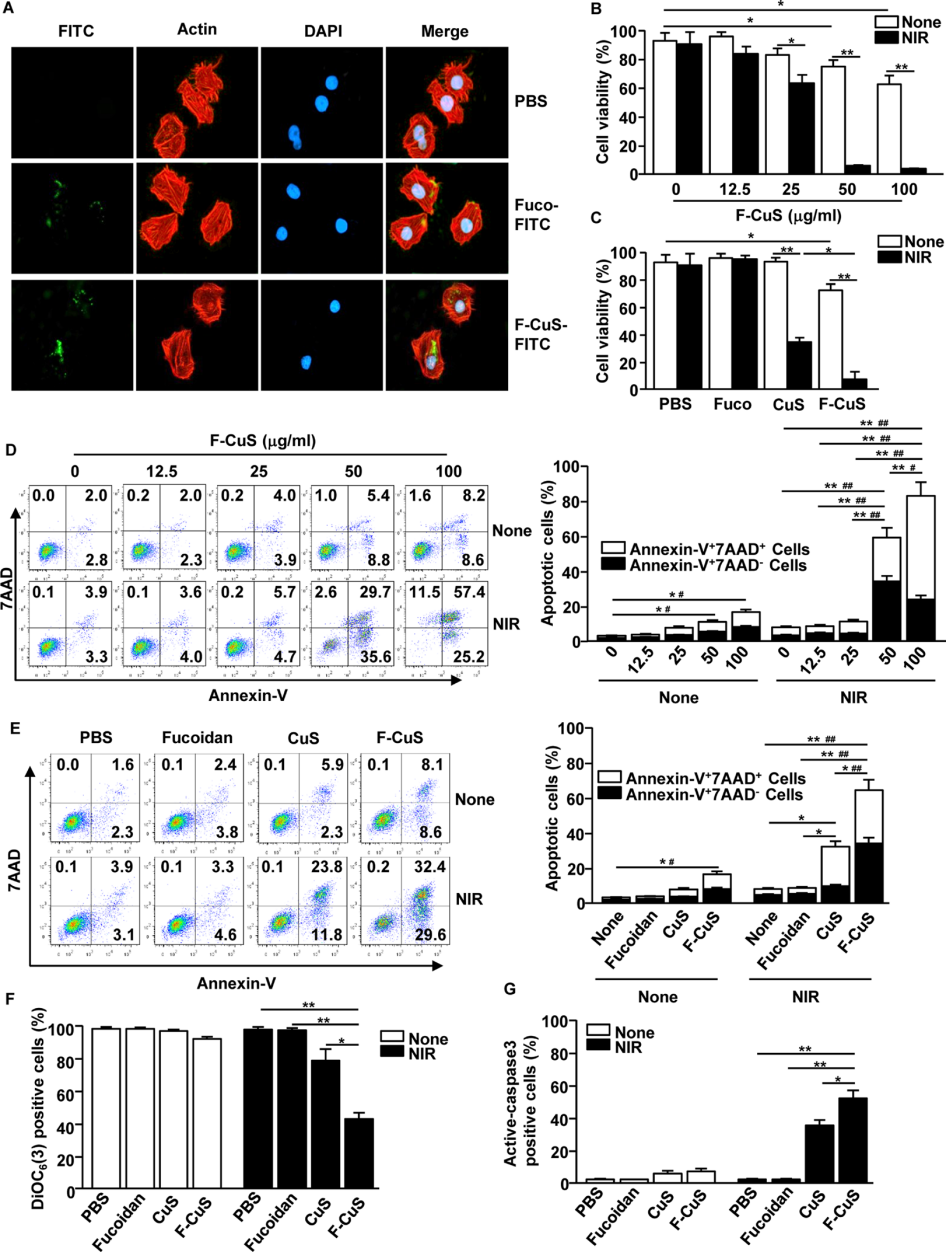
F-CuS-induced apoptosis of HeLa cells (**A**) HeLa cells (2 × 10^5^) were incubated with fucoidan-FITC and F-CuS-FITC for 2 h. The cells were then stained with β-actin and 4′,6-Diamidino-2-Phenylindole, Dihydrochloride (DAPI). The FITC expression levels were measured with a confocal microscope. (**B**) HeLa cells were treated with an indicated concentration of F-CuS, and the cells were irradiated for 5 min with an 808 nm laser. Cell viability was analyzed by MTT assay. (**C**) MTT analysis of fucoidan (75 ng/mL), CuS (200 μg/mL), and F-CuS (50 μg/mL) -treated HeLa cells with or without NIR irradiation. (**D**) Dose-dependent apoptotic effect of F-CuS with or without NIR irradiation was analyzed by annexin-V and 7AAD staining (left panel). Mean percentages of early-apoptotic cells (annexin-V^+^7AAD^–^ cells) and late-apoptotic/necrotic cells (annexin-V^+^7AAD^+^ cells) are shown (right panel). ^#^*p* < 0.05, ^##^*p* < 0.01 for early-apoptotic cells; ^*^p < 0.05, ^**^p < 0.01 for late-apoptotic/necrotic cells. (**E**) Apoptosis of HeLa cells following treatment with fucoidan, CuS, and F-CuS with or without NIR irradiation was analyzed through flow cytometry. The mean percentages of early-apoptotic cells (annexin-V^+^7AAD^–^ cells) and late-apoptotic/necrotic cells (annexin-V^+^7AAD^+^ cells) are shown (right panel). ^#^*p* < 0.05, ^##^*p* < 0.01 for early-apoptotic cells; ^*^p < 0.05, ^**^p < 0.01 for late-apoptotic/necrotic cells. (**F**) Mitochondria permeability was measured by DiOC_6_(3) reduction in flow cytometry; ^*^p < 0.05, ^**^p < 0.01. (**G**) Activation of caspase-3 was analyzed by flow cytometry, and the mean percentages of positive cells are shown; ^*^p < 0.05, ^**^p < 0.01. All data are representative of, or the average of, analyses of six independent samples (i.e., two samples per experiment, three independent experiments).

Since NPs generally exhibit improved drug delivery *in vitro* and *in vivo*, one can speculate that fucoidan can be effectively delivered to the intracellular environment. To quantify the amount of coated fucoidan, thermogravimetric analysis (TGA) was performed [33, 34]; we found that the estimated mass of fucoidan compared to the total mass of F-CuS was ~10 wt% (Supplementary Figure 4). Next, we also examined whether F-CuS is more efficient in delivering fucoidan to cancerous cells than fucoidan alone. We prepared fluorescein isothiocyanate (FITC)-labelled fucoidan and F-CuS, separately, to detect by fluorescence microscopy the intracellular uptake of the two materials. HeLa cells were treated with 75 ng/mL fucoidan-FITC or 50 μg/mL F-CuS-FITC. Here, the concentration of fucoidan was set to the estimated amount of fucoidan coated onto the working concentration of F-CuS (i.e., 50 μg/mL) based on TGA data. Two hours post treatment, we characterized the intracellular uptake of fucoidan and observed that FITC fluorescence signals as per confocal laser scanning microscopy (Figure 3A) and flow cytometry (Supplementary Figure 5) were substantially higher in F-CuS-FITC-treated HeLa cells than those treated with fucoidan-FITC (Figures 3A and Supplementary Figure 5). These data suggest that NP formulation may play an important role in the intracellular uptake of fucoidan into cancerous cells.

To this end, we examined the anti-cancer effect of F-CuS. First, HeLa cells were incubated with an indicated concentration of F-CuS for 2 h and subsequently irradiated for 5 min using an 808 nm laser with a power intensity of 2 W/cm^2^. Cell viability decreased moderately (60–80%) following treatments with 50 and 100 μg/mL F-CuS without NIR irradiation (Figure 3B). Interestingly, NIR irradiation of F-CuS-treated HeLa cells reduced their viability to 60%, even in the case of the 25 μg/mL F-CuS treatment. Furthermore, NIR treatments with higher concentrations of F-CuS (i.e., 50 and 100 μg/mL) showed almost complete killing of the cells (Figure 3B).

Next, we evaluated whether coating CuS with fucoidan is beneficial in killing HeLa compared to CuS without the coating of fucoidan. Since LbL coatings onto CuS with additional materials, such as PAH and fucoidan, change the total mass of substances in solution, we fixed the hyperthermia parameter by adjusting the concentration of CuS (200 μg/mL) and F-CuS (50 μg/mL) to reach the same temperature (50°C), under identical irradiation conditions (Supplementary Figure 6). Here, it should be noted that the concentration of CuS was higher than that of F-CuS; this is because unlike F-CuS, CuS cannot be isolated by centrifugation. Thus, the freeze-dried weight containing residual chemicals was used to back-calculate the concentration of crude CuS. In addition, the concentration of fucoidan alone (Fuco) was set to 75 ng/mL—the estimated amount of fucoidan coated onto the working concentration of F-CuS. HeLa cells incubated with Fuco, CuS, or F-CuS for 2 h were again treated with NIR under identical conditions, and the resulting cell viability was determined. First, it was immediately apparent that the presence of CuS upon NIR irradiation resulted in significant cell death (Figure 3C) due to hyperthermia. Interestingly, under NIR irradiation, F-CuS-treated cells exhibited three times lower cell viability than CuS-treated ones; this can be interpreted as indicative of the synergistic effect of the chemo-agent and elevated temperature. Additionally, consistent with the data regarding the intracellular uptake of Fuco and F-CuS (Figure 3A), Fuco was found to have failed in inducing cell death, as Fuco cannot be effectively delivered into the cells.

### Synergistic anti-cancer effects of F-CuS by fucoidan and PTT

For successful tumor elimination, the induction of apoptosis is essential since apoptotic cells promote the activation of tumor antigen-specific immune responses, including cytotoxic T lymphocyte activation [21, 22]. Apoptosis is characterized by DNA fragmentation, mitochondrial damage, and phosphatidylserine exposure in the cells [35]. Fucoidan has already been found to induce apoptosis in both HeLa and A549 cells, which appear in different human organs (i.e., the cervix and lung, respectively) [8, 36, 37]. Before we evaluated the apoptotic effect of F-CuS with laser irradiation, we examined cytotoxic activity in the RAW cells (a murine macrophage cell line) and found that no cytotoxic effect by F-CuS treatment for 24 h (Supplementary Figure 7). Therefore, we examined annexin-V and 7AAD (7- Aminoactinomycin D) staining to determine apoptosis and necrosis, respectively. The agreed-upon MTT assay for HeLa—namely, 50 and 100 μg/mL F-CuS treatments without NIR irradiation—induced slight increases in early apoptosis and late apoptosis/necrosis (Figure 3D); NIR irradiation promoted dramatic increases in apoptotic cells in the 50 μg/mL F-CuS treatment (Figure 3D). Importantly, under NIR irradiation, 100 μg/mL F-CuS induced great increases in late apoptosis/necrosis but showed an obviously low effect in the promotion of early apoptosis compared to the 50 μg/mL F-CuS treatment (Figure 3D). NIR irradiation in the CuS-treated cells also induced a significant elevation of late apoptosis/necrosis compared to nonirradiated controls but the early-apoptotic cell percentages were markedly lower than those of the F-CuS-treated ones (Figure 3E). Consistent with the F-CuS effect in HeLa cells, A549 cells also showed dramatic increases in early and late apoptosis following treatment with F-CuS and NIR irradiation (Supplementary Figure 8). However, we found that Fuco could not induce apoptosis in either HeLa or A549 cells at a given concentration (i.e., 75 ng/mL). We assume that these findings were due to the low concentration of Fuco. According to previous reports, 10–200 μg/mL fucoidan effectively induces cell apoptosis [8, 10, 36–38], but we used a mere 75 ng/mL of fucoidan coating NPs by LbL. It should be noted that F-CuS without NIR irradiation induced a slightly higher rate of apoptosis in HeLa and A549 cells, compared to that of Fuco at the same dose. This elevated apoptotic effect of F-CuS may be due to the improved cellular uptake of fucoidan, since F-CuS formulation led to higher amounts of intracellular fucoidan delivery than did Fuco, even at a relatively low concentration [39].

To further determine the apoptotic effect of F-CuS, we studied the apoptosis signaling pathway, including the change of mitochondrial permeability, mitochondrial membrane potential, and caspase-3 activation. NIR irradiation in F-CuS-treated HeLa cells induced marked increases in mitochondrial permeability (Figures 3F and Supplementary Figure 9A) and caspase-3 activity (Figures 3G and Supplementary Figure 9B) and decreased mitochondrial membrane potential (Supplementary Figure 9C) compared with nonirradiated or Fuco controls. Such increased effects of apoptotic activities were significantly stronger than those in the CuS-induced samples (Figures 3F, G, and Supplementary Figure 9). Consistent with HeLa cell data, A549 cells also dramatically increased the mitochondrial permeability and caspase-3 activity and decreased the mitochondrial membrane potential after treatment with F-CuS combined with laser irradiation (Supplementary Figure 10). The F-CuS was engineered to have pH-responsive drug delivery nanostructures, as fucoidan is gradually released from the system when NPs are taken up by cancer cells in an acidic environment [31]. At such a low pH, fucoidan sulfates become protonated, and electrostatic interaction between PAH and fucoidan consequently weakens [32]. In this pH-responsive manner, fucoidan will be in its free form to induce apoptosis in the intracellular areas of cancer cells. To confirm, we have measured zeta potential data of the sample at different pH conditions, and as pH became lower, the sample showed positive measurement due to the release of negatively charged fucoidan (Supplementary Figure 11). This explains why CuS did not efficiently promote apoptosis in HeLa and A549 cells, compared to F-CuS. In addition, the effect of fucoidan in promoting mitochondria-dependent apoptosis in cancer cells has been already been well determined [6, 8–10]. Thus, these data suggest that F-CuS promotes the enhanced intracellular delivery of fucoidan and NIR irradiation in F-CuS-treated cells, inducing apoptosis in cancer cells through the fucoidan-induced apoptotic effect and the hyperthermic effect.

### F-CuS-mediated chemo–photothermal therapy *in vivo*

**Figure 4:**
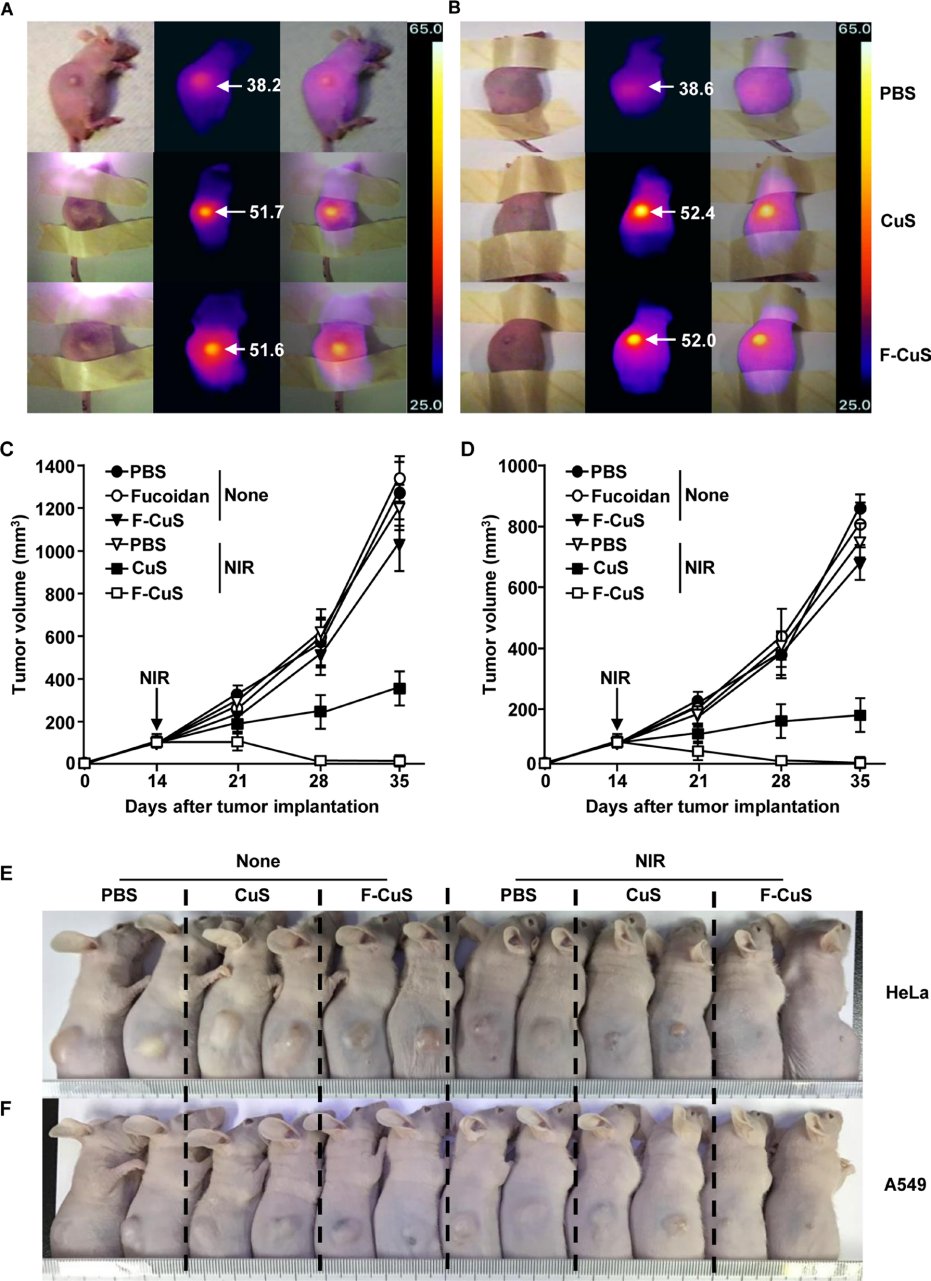
Chemo–photothermal therapy by F-CuS Nude mice were injected *s.c.* with 5 × 10^6^ HeLa cells and 5 × 10^6^ A549 cells. Once tumors were measured to be ~5.0 mm (after 14 d), the mice were treated *i.t.* with 4 μg/kg fucoidan, 10 mg/kg CuS, or 2.5 mg/kg F-CuS. Two hours after treatment, the mice were irradiated for 5 min with an 808 nm laser at 2 W/cm^2^. (**A** and **B**) Thermal image of HeLa tumor mice (A) and A549 tumor mice (B) are shown after NIR irradiation. (**C** and **D**) Tumor volumes of mice injected with HeLa (**C**) and A549 (D) were measured. (**E** and **F**) Tumor masses in the mice are shown after the mice were sacrificed on day 35 of HeLa (E) and A549 (F) cell xenograft. All data are representative of, or the average of, analyses of six independent samples (i.e., two samples per experiment, three independent experiments).

Our data, where F-CuS treatment with NIR irradiation induced apoptosis in HeLa and A549 cells, encouraged us to examine the therapeutic effect of F-CuS against tumors in a mouse model. Nude mice were subcutaneously (*s.c.*) xenografted with HeLa or A549 cells. Once tumors were well established 14 d after cell implantation, the mice received an intratumoral (*i.t.*) injection of PBS, 4 μg/kg fucoidan, 10 mg/kg CuS, or 2.5 mg/kg F-CuS. Two hours after administration, PBS, CuS, and F-CuS-treated mice were irradiated for 5 min with an 808 nm laser (2 W/cm^2^) and were monitored for tumor growth. As shown in the photothermal images in Figure 4A and 4B, there was a substantial NIR irradiation-induced temperature increase in the CuS and F-CuS-treated HeLa and A549 tumors from 25°C to 51°C or higher, while PBS-injected tumors showed slight skin-temperature increases of up to 38°C under NIR irradiation. After NIR irradiation, the size of each HeLa and A549 tumor was gradually reduced in the F-CuS-treated mice (Figure 4C and 4D). On day 35 of tumor injection, the HeLa and A549 tumors had almost disappeared with F-CuS treatment combined with NIR irradiation (Figures 4E, F, and Supplementary Figure 12). Although CuS-treated mice with HeLa or A549 tumors also showed remarkably inhibited tumor growth compared to PBS or Fuco-treated controls, the F-CuS treatment promoted a more efficient therapeutic effect than did the CuS treatment (Figures 4E, F, and Supplementary Figure 12). During the HeLa and A549 tumor therapy featuring the use of F-CuS and NIR irradiation, we also measured changes in body weights to evaluate the cytotoxic effect in the mice; we found that neither CuS nor F-CuS with NIR irradiation altered body weights when compared to the PBS- or fucoidan-treated mice (Supplementary Figure 13). In addition, the F-CuS treatment did not promote immune cell infiltration in the peripheral tissues, thus indicating that F-CuS treatment did not induce tissue inflammation (Supplementary Figure 14). Thus, these *in vivo* results support the assertion that the use of F-CuS in combination with NIR irradiation efficiently promotes therapeutic effects against HeLa and A549 tumors in mice, without any adverse side effects (e.g., loss of body weight or inflammation of peripheral tissues).

### Therapeutic effect of F-CuS against multi-drug-resistant K562 cells

Since K562 cells are well known as multidrug-resistant suspension cells and fucoidan resistant [6, 40], we next examined the effect of F-CuS in inducing cell death, via NIR irradiation and the intracellular delivery of fucoidan. K562 cells were treated with FITC-conjugated fucoidan or F-CuS, and 2 h later we measured cellular FITC signals. F-CuS-treated cells exhibited much higher intracellular fluorescence FITC intensity than the controls, including cells treated with Fuco (Supplementary Figure 15). These data give us confidence that F-CuS could be a suitable multi-therapeutic material against K562 cells.

**Figure 5:**
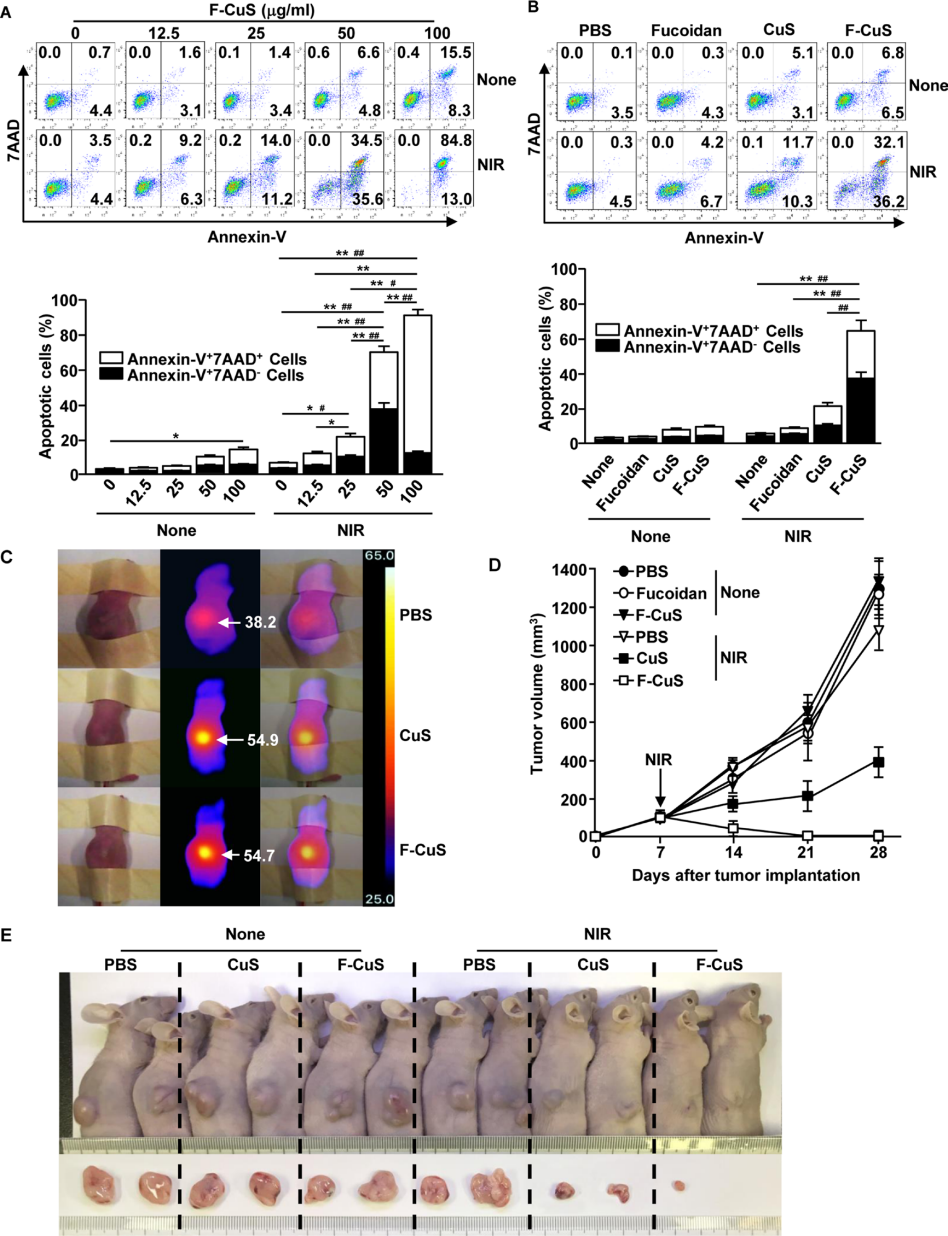
F-CuS-mediated chemo–photothermal therapy against multi-drug-resistant K562 cells (**A**) K562 cells (2 × 10^5^) were treated with an indicated dose of F-CuS for 2 h; cells were irradiated for 5 min with an 808 nm laser at 2.5 W/cm^2^. Apoptosis of K562 cells was analyzed by annexin-V & 7AAD staining (upper panel). (B) K562 cells were treated with 75 ng/mL fucoidan, 200 μg/mL CuS, or 50 μg/mL F-CuS for 2 h, and then the cells were irradiated for 5 min with an 808 nm laser at 2.5 W/cm^2^. Apoptotic K562 cells are shown (upper panel). In (A) and (**B**), the mean percentages of early-apoptotic cells (annexin-V^+^7AAD^–^ cells) and late-apoptotic/necrotic cells (annexin-V^+^7AAD^+^ cells) are shown (right panel). ^#^*p* < 0.05, ^##^*p* < 0.01 for early-apoptotic cells; ^*^p < 0.05, ^**^p < 0.01 for late-apoptotic/necrotic cells (lower panel). (**C** to **E**) Nude mice were injected *s.c.* with 5 × 10^6^ K562 cells. Once tumors measured to be ~5.0 mm (after 14 d), the mice were treated *i.t.* with 4 μg/kg fucoidan, 10 mg/kg CuS, or 2.5 mg/kg F-CuS. Two hours after treatment, the mice were irradiated for 5 min with an 808 nm laser at 2.5 W/cm^2^. (C) Thermal images of mice are shown after NIR irradiation. (D) Tumor volumes are shown. (E) Tumor masses in the mice are shown on day 28 of NIR irradiation. Data are representative of analyses of four independent samples (i.e., two mice per experiment, two independent experiments).

Next, we evaluated the apoptotic effect of F-CuS with or without NIR irradiation in K562 cells. Consistent with the findings from other cell lines (HeLa and A549), 50 or 100 μg/mL of F-CuS induced considerable increases in apoptosis in K562 cells (Figure 5A). Moreover, cells treated with 50 μg/mL of F-CuS showed substantial increases in early apoptosis and late apoptosis/necrosis under 5 min of 808 nm laser irradiation (2.5 W/cm^2^) (Figure 5A). However, 100 μg/mL F-CuS with NIR irradiation induced late apoptosis/necrosis without early apoptosis. In addition, NIR irradiation in 50 μg/mL F-CuS-treated K562 cells promoted significantly higher early-apoptosis and late-apoptosis/necrosis cell populations, compared to CuS-treated K562 cells (Figure 5B). Furthermore, F-CuS and laser irradiation promoted increases in the mitochondria permeability and caspase-3 activation and decreases in the mitochondrial membrane potential (Supplementary Figure 16). Thus, these data suggest that F-CuS administration in conjunction with NIR irradiation also triggers the promotion of apoptosis in multidrug-resistant cells (i.e., K562).

We then examined the elimination of K562 cells in the mouse model. Nude mice were *s.c.* xenografted with K562 cells. Two weeks after tumor implantation, the mice were treated with an *i.t.* injection of PBS, 40 μg/kg fucoidan, 10 mg/kg CuS, or 2.5 mg/kg F-CuS for 2 h and irradiated for 5 min with an 808 nm laser (2.5 W/cm^2^). As shown in Figure 5C, NIR irradiation caused remarkable increases in temperature in the CuS and F-CuS-treated tumors, whereas negligible temperature increases were observed in the PBS-treated mice. Moreover, tumors completely disappeared in the F-CuS-treated mice on day 28 of tumor injection, whereas tumors were still apparent in the CuS-treated mice—although they were somewhat smaller than those of other controls (Figure 5D and 5E). The weight of K562 tumor mass also significantly decreased through treatment of F-CuS combined with laser irradiation (Supplementary Figure 17). Furthermore, treatment with F-CuS and NIR irradiation did not induce loss of body weight in the mice during treatment (Supplementary Figure 18).

Previous research has shown that fucoidan does not promote apoptosis in K562 cells. Moreover, the apoptosis-signaling pathway in K562 cells—including the ERK, JNK, and caspase-signaling pathways—was found not to be activated by fucoidan [6]. In the present study, we found that F-CuS promoted considerable apoptosis in K562 cells, even in the absence of NIR irradiation. Compared to HeLa and A549 cells (Figures 3A and Supplementary Figure 5), K562 cells did not take up fucoidan intracellularly (Supplementary Figure 15); this suggests that drug resistance against fucoidan-induced apoptosis may be due to the failure to take up fucoidan intracellularly. In the present study, F-CuS efficiently delivered fucoidan into the intracellular areas of K562 cells, resulting in induced cell apoptosis; this may be due to the intracellular delivery of fucoidan by NPs. Therefore, we concluded that the chemo- and phototherapeutic effects of F-CuS were also applicable to multi-drug-resistant K562 cells. In future research, we will investigate the apoptotic effect of F-CuS without NIR irradiation and whether a high concentration of F-CuS can induce apoptosis in K562 cells and activate apoptotic signaling.

## MATERIALS AND METHODS

### Materials and measurement

All chemicals were purchased from Sigma Aldrich Chemical, Inc. (St. Louis, MO, USA), unless otherwise specified. Sodium Citrate (C_6_H_5_O_7_Na_3_·2H_2_O) was purchased from the DC Chemical Co. Ltd (Seoul, South Korea). Field emission transmission electron microscopy (FE-TEM) images were taken with a JEM-2100F transmission electron microscope (JEOL; Tokyo, Japan). To determine the chemical composition of the nanoparticles, EDS (JXA-8530F; JEOL, Tokyo, Japan) was used. FT-IR spectra were measured with a Spectrum GX (PerkinElmer Inc.; Waltham, MA, USA). Power X-ray diffraction graphs were measured by an X’Pert-MPD System (Philips; Amsterdam, Netherlands). Dynamic light scattering and zeta potential measurements were taken with an ELS-8000 (Otsuka Electronics Co. Ltd.; Osaka, Japan). A thermogravimetric analyzer (TGA 7, Pyris 1; PerkinElmer Inc.; Waltham, MA, USA) was used to analyze the fucoidan coating and its concentration. UV-vis absorption spectra were recorded UV-Visible spectrophotometer (Beckman Coulter; Fullerton, CA, USA). A fiber-coupled continuous-wave diode laser (808 nm, 10 W) was purchased from Changchun New Industries Optoelectronics Technology Co., Ltd. (Changchun, China). Thermographic images were taken using a FLIR ONE (FLIR Systems, Wilsonville, OR, USA). Intracellular fluorescence imaging was observed using a Leica laser scanning confocal microscope (Leica Microsystems; Wetzlar, Germany). Flow cytometry analysis data were obtained by using a BD LSR Fortessa (Becton Dickinson; Coppelle, TX, USA).

### Preparation of F-CuS

Citrate-stabilized copper sulfide nanoparticles were synthesized according to the previously published report [28]. To coat CuS with PAH [29], we added crude CuS solution (50 mL) under sonication to a beaker containing 50 mL of PAH solution (Mw = 17500, 2 mg/mL) dropwise manner; the mixture was stirred for 4 h. To isolate PAH-CuS, the mixture was centrifuged at 19,000 rpm for 5 h; the dark green pellet was resuspended in 50 mL DI water. The fucoidan coating on PAH-CuS was performed according to the literature, with slight modification [30]. PAH-CuS (50 mL) was added dropwise to 50 mL of fucoidan solution (1 mg/mL) under vigorous stirring. Again, centrifugation was performed to isolate the F-CuS pellet, which was resuspended in a stock solution (5 mg/mL). F-CuS used throughout all experiments herein were from single batch. FITC-conjugated F-CuS was prepared according to the reference with the slight modification [41, 42]. FITC (0.01M) in ethanol (1 mL) was stirred for 24 h at 30°C. Centrifuged F-CuS sample and triethylamine were added to the prepared FITC solution, and then, the solution was stirred for another 24 h at 50°C. FITC-conjugated F-CuS sample was centrifuged at 19,000 rpm for 5 h to remove unreacted FITC.

### Mice

Six-week-old BALB/c nu/nu (nude) mice were obtained from the Shanghai Public Health Clinical Center and kept under pathogen-free conditions. The mice were maintained at a controlled temperature of 20–22°C, humidity of 50–60%, and 12:12-h lighting, with free access to standard rodent chow and water. All experiments were carried out in agreement with the guidelines of the Institutional Animal Care and Use Committee at the Shanghai Public Health Clinical Center. The protocol was approved by the Committee on the Ethics of Animal Experiments for the Shanghai Public Health Clinical Center (Mouse Protocol Number: SYXK-2010-0098). Mice were euthanatized through CO_2_ inhalation, and all efforts were made to minimize suffering.

### Cells

HeLa, A549, and K562 cells were provided by the American Type Culture Collection (Rockwile, MD, USA). The cells were cultured in RPMI 1640 medium (Gibco; Paisley, UK) that was supplemented with 10% fetal calf serum (Gibco; Paisley, UK), 120 mg/L penicillin, and 200 mg/L streptomycin at 37°C and 5% CO_2_.

### *In vitro* photothermal treatment

HeLa and A549 cells (1 × 10^5^) were seeded into a 24-well plate for 24 h; they were then treated with fucoidan, CuS, or F-CuS for 2 h, and the cells were irradiated for 5 min with an 808 nm laser at 2 W/cm^2^. In the case of the K562 cells, the cells were irradiated for 5 min at 2.5 W/cm^2^.

### MTT assay

After 24 h of incubation, 100 mL of finely resuspended control and heat shock-treated cells were transferred to 96 flat-bottomed microtiter plates. Then, 10 μL of freshly prepared MTT solution PBS was added to each well and incubated for 4 h. Finally, 50 μL of MTT lysis solution (20% sodium dodecyl sulfate W/V and 50% dimethyl formamide V/V) was added to each well and incubated overnight. Absorbance was read at 620 nm, using an enzyme-linked immunosorbent assay (ELISA) reader (Labsystems Multiskan; Roden, Netherlands).

### Annexin-V and 7AAD staining

Cultured cells were stained with annexin V-FITC and 7AAD (7-Aminoactinomycin D) in 100 μL of binding buffer for 15 min at room temperature (RT). After 400 μL of binding buffer was added, the cells were analyzed by flow cytometry using a FACS Aria II (Becton Dickinson; San Diego, CA, USA).

### Mitochondrial permeability assay

HeLa and K562 cells (2 × 10^6^/mL) were treated with 40 nM of 3,3′-dihexyloxacarbocyanine iodide [DiOC_6_(3); Sigma-Aldrich, St. Louis, MO, USA] for 30 min at 37°C and then washed with PBS. Stained cells were analyzed by flow cytometry.

### Analysis of caspase-3 activation

The activation of caspase-3 was determined with a CaspGLOW^TM^ Red Active Caspase-3 Staining Kit (BioVision; Milpitas, CA, USA), according to the manufacturer’s protocols. Briefly, the cells were incubated with active caspase-3 staining reagent for 30 min at RT. After washing with washing buffer, the cells were resuspended with 500 μL PBS and analyzed by flow cytometry.

### *In vivo* photothermal treatment

Nude mice were *s.c.* injected with 5 × 10^6^ HeLa, A549, or K562 cells. Once tumors at their longest dimension reached a size of ~5.0 mm, mice were randomized into six treatment groups: PBS, fucoidan, F-CuS, PBS with laser treatment, CuS NPs with laser treatment, and F-CuS with laser treatment. Each NP type was *i.t.* injected into the mice. Two hours after injection, an 808 nm NIR laser was applied to irradiate tumors under a power intensity of 2 W/cm^2^ (HeLa and A549) or 2.5 W/cm^2^ (K562). The temperature was recorded using the infrared camera FLIR One Thermal imaging system (FLIR; Wilsonwille, OR, USA). Tumor volume was calculated using the formula V ¼ 1/2 (L/S2), where L is the longest dimension and S is the shortest dimension.

### Statistical analysis

All statistical analysis results are expressed as the mean ± standard error of the mean. The statistical significance of differences between experimental groups was calculated using analysis of variance, and either a Bonferroni posttest or an unpaired Student’s *t*-test. All *p*-values < 0.05 were considered significant.

## CONCLUSIONS

To derive an enhanced therapy by which to cure cancer, we prepared nanoparticles *via* a LbL technique, where NIR-absorbing material was coated with a natural anti-cancer agent. We demonstrated that our material can induce apoptosis in cancer cells by combining the fucoidan-mediated anti-cancer effect and the NIR-induced hyperthermic effect; this combination consequently eliminated multiple tumors in live mouse models. Thus, this chemo-photothermal therapy, which uses NIR-absorbing NPs and a biologically active component, will be a promising candidate in the fields of material sciences and cancer nanotechnology.

## SUPPLEMENTARY MATERIALS FIGURES



## References

[1] Wang AZ, Langer R, Farokhzad OC (2012). Nanoparticle delivery of cancer drugs. Annu Rev Med.

[2] Cunha L, Grenha A (2016). Sulfated Seaweed Polysaccharides as Multifunctional Materials in Drug Delivery Applications. Mar Drugs.

[3] Durig J, Bruhn T, Zurborn KH, Gutensohn K, Bruhn HD, Beress L (1997). Anticoagulant fucoidan fractions from Fucus vesiculosus induce platelet activation in vitro. Thromb Res.

[4] Hayashi K, Lee JB, Nakano T, Hayashi T (2013). Anti-influenza A virus characteristics of a fucoidan from sporophyll of Undaria pinnatifida in mice with normal and compromised immunity. Microbes Infect.

[5] Jin JO, Park HY, Xu Q, Park JI, Zvyagintseva T, Stonik VA, Kwak JY (2009). Ligand of scavenger receptor class A indirectly induces maturation of human blood dendritic cells via production of tumor necrosis factor-alpha. Blood.

[6] Jin JO, Song MG, Kim YN, Park JI, Kwak JY (2010). The mechanism of fucoidan-induced apoptosis in leukemic cells: involvement of ERK1/2, JNK, glutathione, and nitric oxide. Mol Carcinog.

[7] Jin JO, Zhang W, Du JY, Wong KW, Oda T, Yu Q (2014). Fucoidan can function as an adjuvant in vivo to enhance dendritic cell maturation and function and promote antigen-specific T cell immune responses. PLoS One.

[8] Boo HJ, Hyun JH, Kim SC, Kang JI, Kim MK, Kim SY, Cho H, Yoo ES, Kang HK (2011). Fucoidan from Undaria pinnatifida induces apoptosis in A549 human lung carcinoma cells. Phytother Res.

[9] Chen S, Zhao Y, Zhang Y, Zhang D (2014). Fucoidan induces cancer cell apoptosis by modulating the endoplasmic reticulum stress cascades. PLoS One.

[10] Zhang Z, Teruya K, Eto H, Shirahata S (2011). Fucoidan extract induces apoptosis in MCF-7 cells via a mechanism involving the ROS-dependent JNK activation and mitochondria-mediated pathways. PLoS One.

[11] Lira MC, Santos-Magalhaes NS, Nicolas V, Marsaud V, Silva MP, Ponchel G, Vauthier C (2011). Cytotoxicity and cellular uptake of newly synthesized fucoidan-coated nanoparticles. Eur J Pharm Biopharm.

[12] Manivasagan P, Oh J (2015). Production of a Novel Fucoidanase for the Green Synthesis of Gold Nanoparticles by Streptomyces sp. and Its Cytotoxic Effect on HeLa Cells. Mar Drugs.

[13] Tengdelius M, Gurav D, Konradsson P, Pahlsson P, Griffith M, Oommen OP (2015). Synthesis and anticancer properties of fucoidan-mimetic glycopolymer coated gold nanoparticles. Chem Commun (Camb).

[14] Ban Q, Bai T, Duan X, Kong J (2017). Noninvasive photothermal cancer therapy nanoplatforms via integrating nanomaterials and functional polymers. Biomater Sci.

[15] Dickerson EB, Dreaden EC, Huang X, El-Sayed IH, Chu H, Pushpanketh S, McDonald JF, El-Sayed MA (2008). Gold nanorod assisted near-infrared plasmonic photothermal therapy (PPTT) of squamous cell carcinoma in mice. Cancer Lett.

[16] Huang N, Wang H, Zhao J, Lui H, Korbelik M, Zeng H (2010). Single-wall carbon nanotubes assisted photothermal cancer therapy: animal study with a murine model of squamous cell carcinoma. Lasers Surg Med.

[17] Espinosa A, Di Corato R, Kolosnjaj-Tabi J, Flaud P, Pellegrino T, Wilhelm C (2016). Duality of Iron Oxide Nanoparticles in Cancer Therapy: Amplification of Heating Efficiency by Magnetic Hyperthermia and Photothermal Bimodal Treatment. ACS Nano.

[18] Guo L, Yan DD, Yang D, Li Y, Wang X, Zalewski O, Yan B, Lu W (2014). Combinatorial photothermal and immuno cancer therapy using chitosan-coated hollow copper sulfide nanoparticles. ACS Nano.

[19] Same S, Aghanejad A, Akbari Nakhjavani S, Barar J, Omidi Y (2016). Radiolabeled theranostics: magnetic and gold nanoparticles. Bioimpacts.

[20] Goel S, Chen F, Cai W (2014). Synthesis and biomedical applications of copper sulfide nanoparticles: from sensors to theranostics. Small.

[21] Opferman JT, Korsmeyer SJ (2003). Apoptosis in the development and maintenance of the immune system. Nat Immunol.

[22] Groh V, Li YQ, Cioca D, Hunder NN, Wang W, Riddell SR, Yee C, Spies T (2005). Efficient cross-priming of tumor antigen-specific T cells by dendritic cells sensitized with diverse anti-MICA opsonized tumor cells. Proc Natl Acad Sci U S A.

[23] Li X, Liu C, Wang S, Jiao J, Di D, Jiang T, Zhao Q, Wang S (2017). Poly(acrylic acid) conjugated hollow mesoporous carbon as a dual-stimuli triggered drug delivery system for chemo-photothermal synergistic therapy. Mater Sci Eng C Mater Biol Appl.

[24] Yuan A, Huan W, Liu X, Zhang Z, Zhang Y, Wu J, Hu Y (2017). NIR Light-Activated Drug Release for Synergetic Chemo-Photothermal Therapy. Mol Pharm.

[25] Zhang L, Su H, Cai J, Cheng D, Ma Y, Zhang J, Zhou C, Liu S, Shi H, Zhang Y, Zhang C (2016). A Multifunctional Platform for Tumor Angiogenesis-Targeted Chemo-Thermal Therapy Using Polydopamine-Coated Gold Nanorods. ACS Nano.

[26] Liao PC, Lieu CH (2005). Cell cycle specific induction of apoptosis and necrosis by paclitaxel in the leukemic U937 cells. Life Sci.

[27] Shin HJ, Kwon HK, Lee JH, Gui X, Achek A, Kim JH, Choi S (2015). Doxorubicin-induced necrosis is mediated by poly-(ADP-ribose) polymerase 1 (PARP1) but is independent of p53. Sci Rep.

[28] Zhou M, Zhang R, Huang M, Lu W, Song S, Melancon MP, Tian M, Liang D, Li C (2010). A chelator-free multifunctional [64Cu]CuS nanoparticle platform for simultaneous micro-PET/CT imaging and photothermal ablation therapy. J Am Chem Soc.

[29] Dominguez GA, Lohse SE, Torelli MD, Murphy CJ, Hamers RJ, Orr G, Klaper RD (2015). Effects of charge and surface ligand properties of nanoparticles on oxidative stress and gene expression within the gut of Daphnia magna. Aquat Toxicol.

[30] Schneider G, Decher G (2004). From Functional Core/Shell Nanoparticles Prepared via Layer-by-Layer Deposition to Empty Nanospheres. Nano Letters.

[31] Manchun S, Dass CR, Sriamornsak P (2012). Targeted therapy for cancer using pH-responsive nanocarrier systems. Life Sci.

[32] Lu KY, Li R, Hsu CH, Lin CW, Chou SC, Tsai ML, Mi FL (2017). Development of a new type of multifunctional fucoidan-based nanoparticles for anticancer drug delivery. Carbohydrate Polymers.

[33] Caratão B, Carneiro E, Sá P, Almeida B, Carvalho S (2014). Properties of Electrospun TiO2 Nanofibers. Journal of Nanotechnology.

[34] Loof D, Hiller M, Oschkinat H, Koschek K (2016). Quantitative and Qualitative Analysis of Surface Modified Cellulose Utilizing TGA-MS. Materials (Basel).

[35] Kerr JF, Wyllie AH, Currie AR (1972). Apoptosis: a basic biological phenomenon with wide-ranging implications in tissue kinetics. Br J Cancer.

[36] Stevan FR, Oliveira MB, Bucchi DF, Noseda Iacomini M, Duarte ME (2001). Cytotoxic effects against HeLa cells of polysaccharides from seaweeds. J Submicrosc Cytol Pathol.

[37] Ye J, Li Y, Teruya K, Katakura Y, Ichikawa A, Eto H, Hosoi M, Hosoi M, Nishimoto S, Shirahata S (2005). Enzyme-digested Fucoidan Extracts Derived from Seaweed Mozuku of Cladosiphon novae-caledoniae kylin Inhibit Invasion and Angiogenesis of Tumor Cells. Cytotechnology.

[38] Hsu HY, Lin TY, Hwang PA, Tseng LM, Chen RH, Tsao SM, Hsu J (2013). Fucoidan induces changes in the epithelial to mesenchymal transition and decreases metastasis by enhancing ubiquitin-dependent TGFbeta receptor degradation in breast cancer. Carcinogenesis.

[39] Soppimath KS, Aminabhavi TM, Kulkarni AR, Rudzinski WE (2001). Biodegradable polymeric nanoparticles as drug delivery devices. J Control Release.

[40] Rumjanek VM, Trindade GS, Wagner-Souza K, de-Oliveira MC, Marques-Santos LF, Maia RC, Capella MA (2001). Multidrug resistance in tumour cells: characterization of the multidrug resistant cell line K562-Lucena 1. An Acad Bras Cienc.

[41] Davis KB, Braasch DA, Pramanik M, Rawlins JW (2014). Use of Fluorescent Probes to Determine Molecular Architecture in Phase Separating Epoxy Systems. Industrial & Engineering Chemistry Research.

[42] Xiao R, Su Y, Feng T, Sun M, Liu B, Zhang J, Lu Y, Li J, Wang T, Zhu L, Hu Q (2017). Monocrotaline Induces Endothelial Injury and Pulmonary Hypertension by Targeting the Extracellular Calcium-Sensing Receptor. J Am Heart Assoc.

