# Supplementary material for: Fucoidan-coated CuS nanoparticles for chemo-and photothermal therapy against cancer
Source: Oncotarget. 2018 Jan 3;9(16):12649–61. doi: 10.18632/oncotarget.23898 (PMC5849162; doi:10.18632/oncotarget.23898)
Supplement: Supplementary file 1 [file oncotarget-09-12649-s001.pdf]

Fucoidan-coated CuS nanoparticles for chemo- and photothermal therapy against cancer

SUPPLEMENTARY MATERIALS

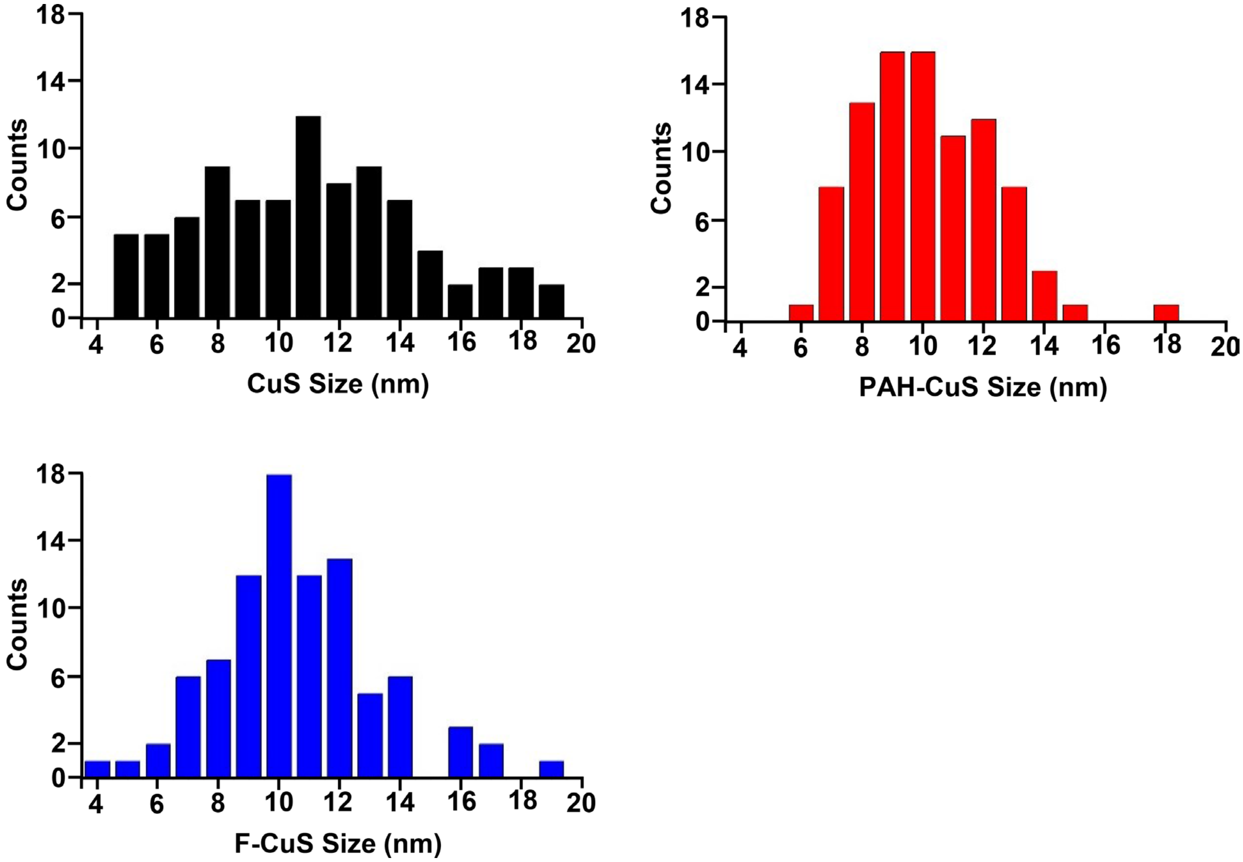

Supplementary Figure 1: TEM corresponding size distribution histogram of each coated nanoparticles: CuS (black), PAH-CuS (red), F-CuS (blue).

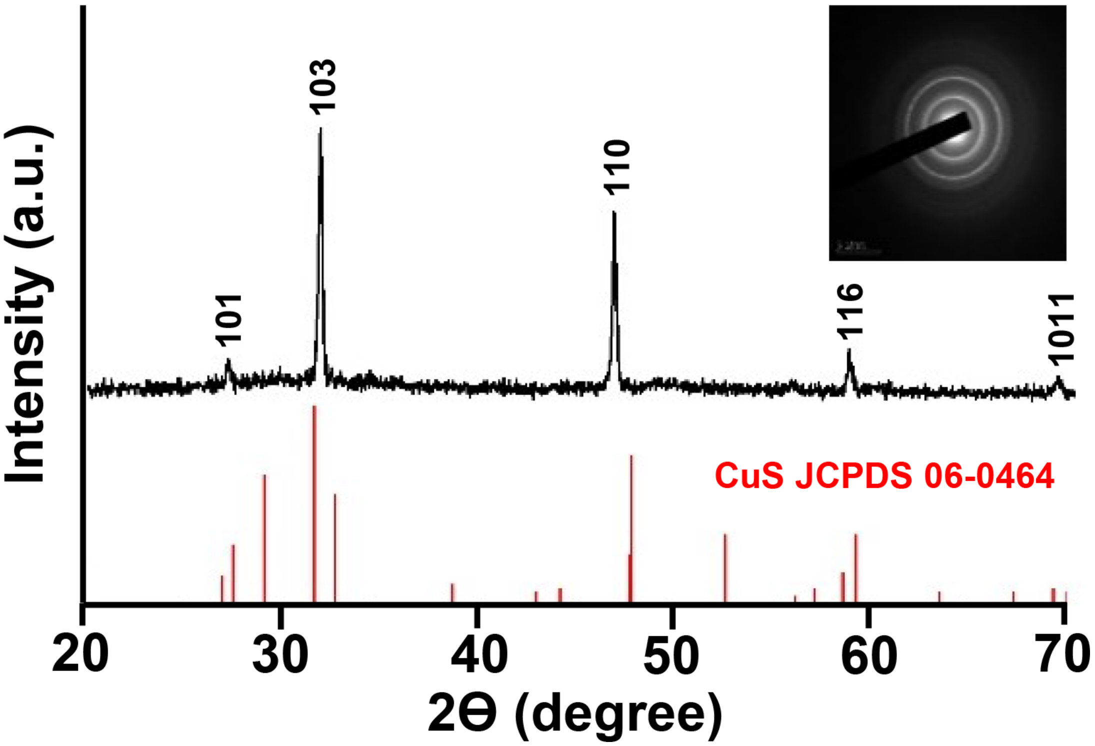

Supplementary Figure 2: XRD pattern and ED pattern(inset) of CuS with the standard JCPDS card 06-0464 of CuS.

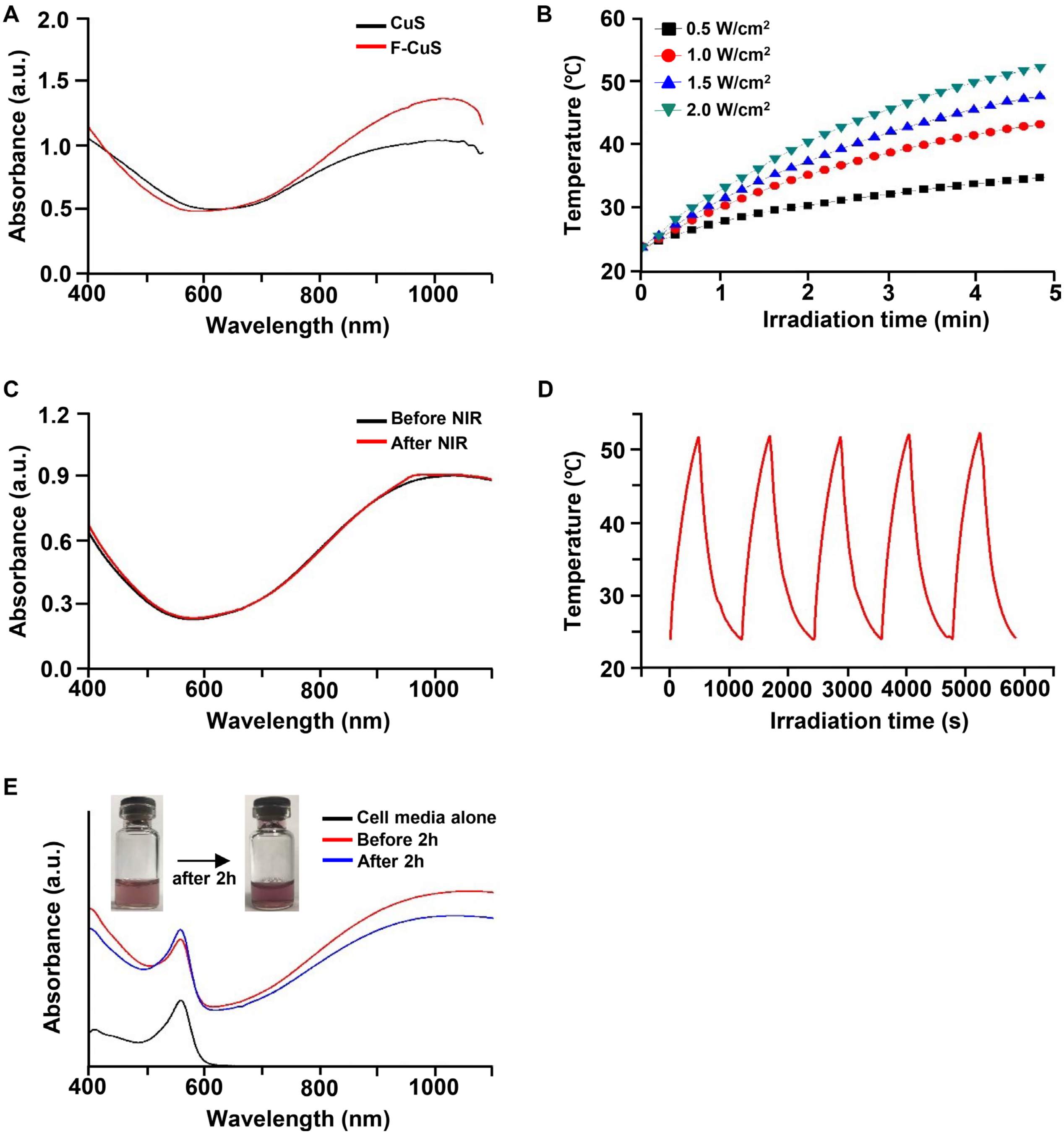

Supplementary Figure 3: (**A**) UV-vis absorption of CuS (black) and F-CuS (red). (**B**) Photothermal heating curves of F-CuS (50 μg/ml) dissolved in water, irradiated for 5 min with an 808 nm laser with indicated power density. (**C**) UV-vis absorption of F-CuS before (black) and after (red) NIR-irradiation. (**D**) Photothermal heating curves of F-CuS (50 μg/mL) during the successful 5 cycles of the laser ON/OFF mode (808 nm laser, 2W/cm^2). (**E**) UV-vis absorption of F-CuS dissoveled in cell media before and after 2 h.

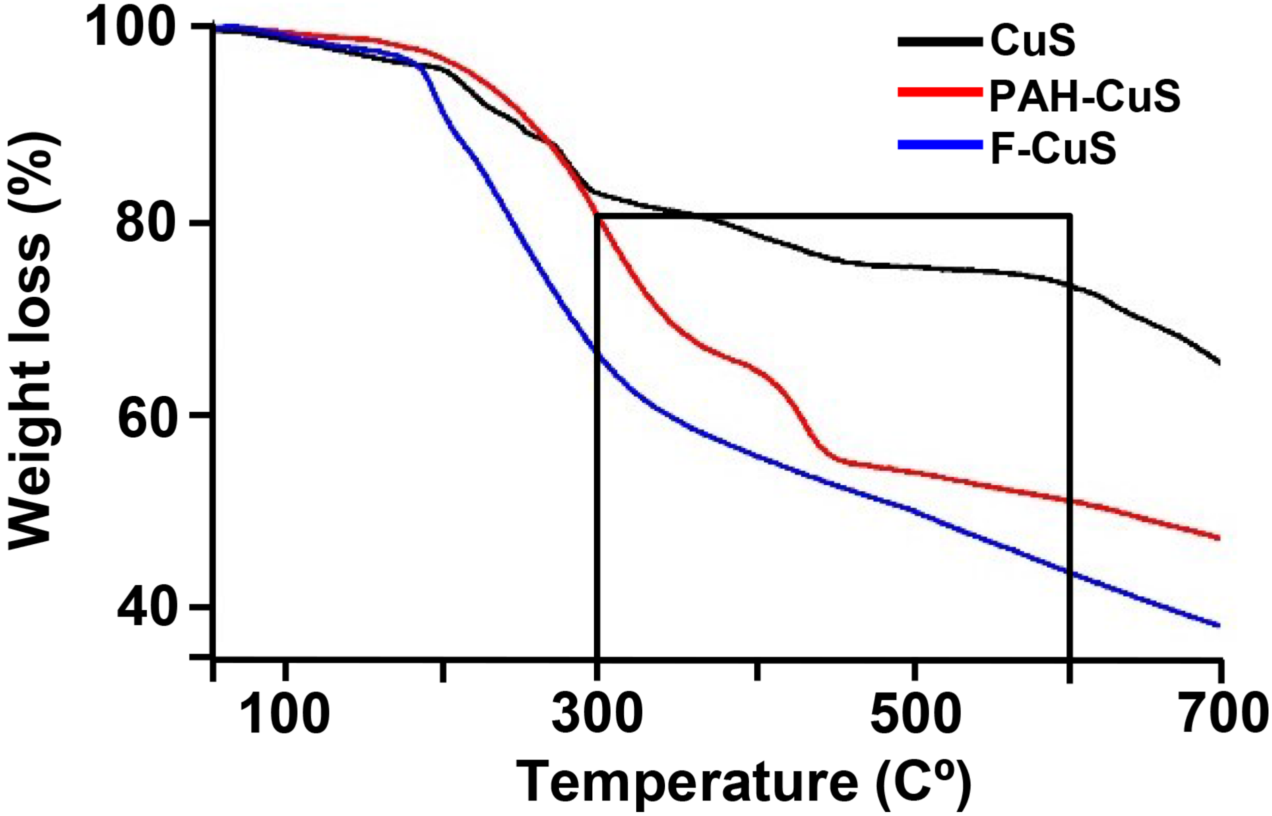

Supplementary Figure 4: TGA curves of each coated nanoparticles: CuS (black), PAH-CuS (red), F-CuS (blue).

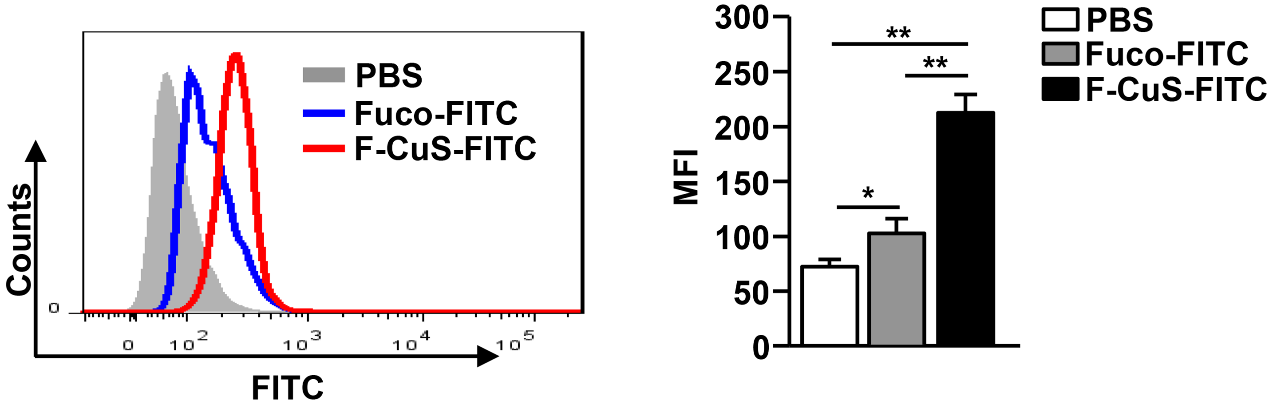

Supplementary Figure 5: Intracellular uptake of fucoidan in HeLa cells by F-CuS. HeLa cells (2×10^5) were incubated with fucoidan-FITC (Fuco-FITC) and F-CuS-FITC for 2 h. The intracellular uptake of fucoidan was analyzed on a flow cytometry (left panel). Mean fluorescence intensity (MFI) of FITC positive cells was shown (right panel), $^{*}p < 0.05$, $^{**}p < 0.01$. Data are representative of or the average of analyses of 6 independent samples (2 samples per experiment, 3 independent experiments).

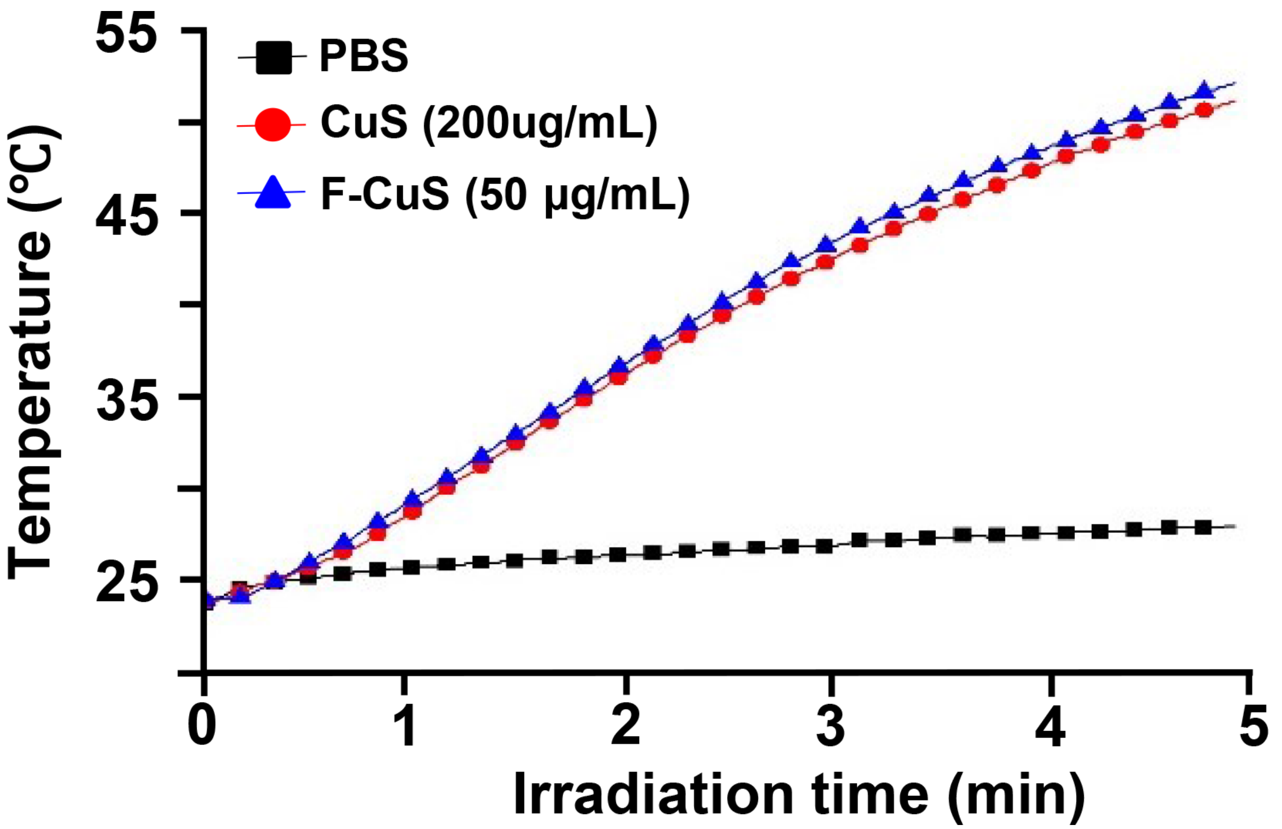

Supplementary Figure 6: Photothermal heating curves of CuS (red) and F-CuS (blue) with different concentration dissolved in PBS irradiated using 808 nm laser with power density of 2 W/cm^2 for 5 min.

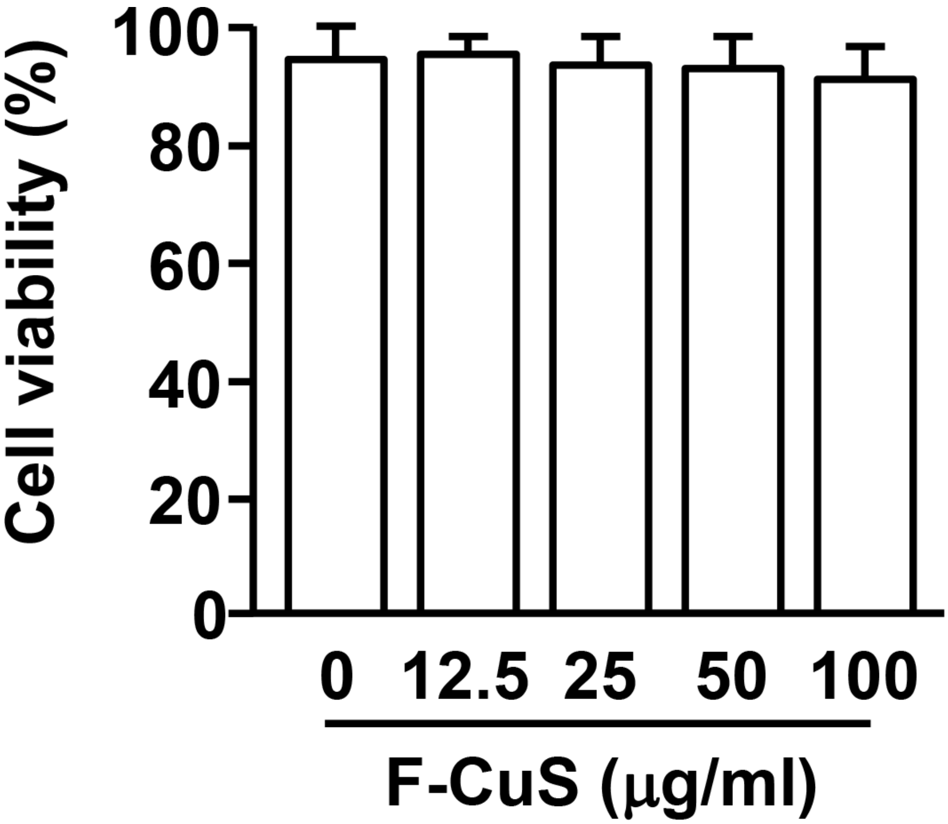

Supplementary Figure 7: RAW cells were treated with an indicated concentration of F-CuS for 24 h. Cell viability was analyzed by MTT assay.

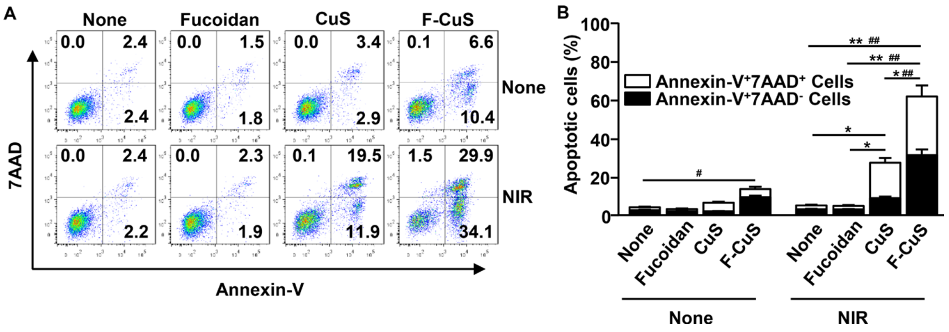

Supplementary Figure 8: Apoptotic effect of F-CuS with laser irradiation against A549 cells. A549 cells (2×10^5) were incubated with fucoidan, CuS and F-CuS-FITC and irradiated with 808 nm laser for 5 min. (**A**) Apoptosis of A549 cells were analyzed by annexin-V and 7AAD staining on a flow cytometry after 24 h of laser irradiation (left panel). (**B**) Mean percentage of early apoptotic cells (Annexin-V+7AAD- Cells) and late apoptotic/necrotic cells (Annexin-V+7AAD+ Cells) were shown (right panel). $^{\#}p < 0.05$, $^{\#\#}p < 0.01$ early apoptotic cells, $^{*}p < 0.05$, $^{**}p < 0.01$ late apoptotic/necrotic cells. Data are representative of or the average of analyses of 6 independent samples (2 samples per experiment, 3 independent experiments).

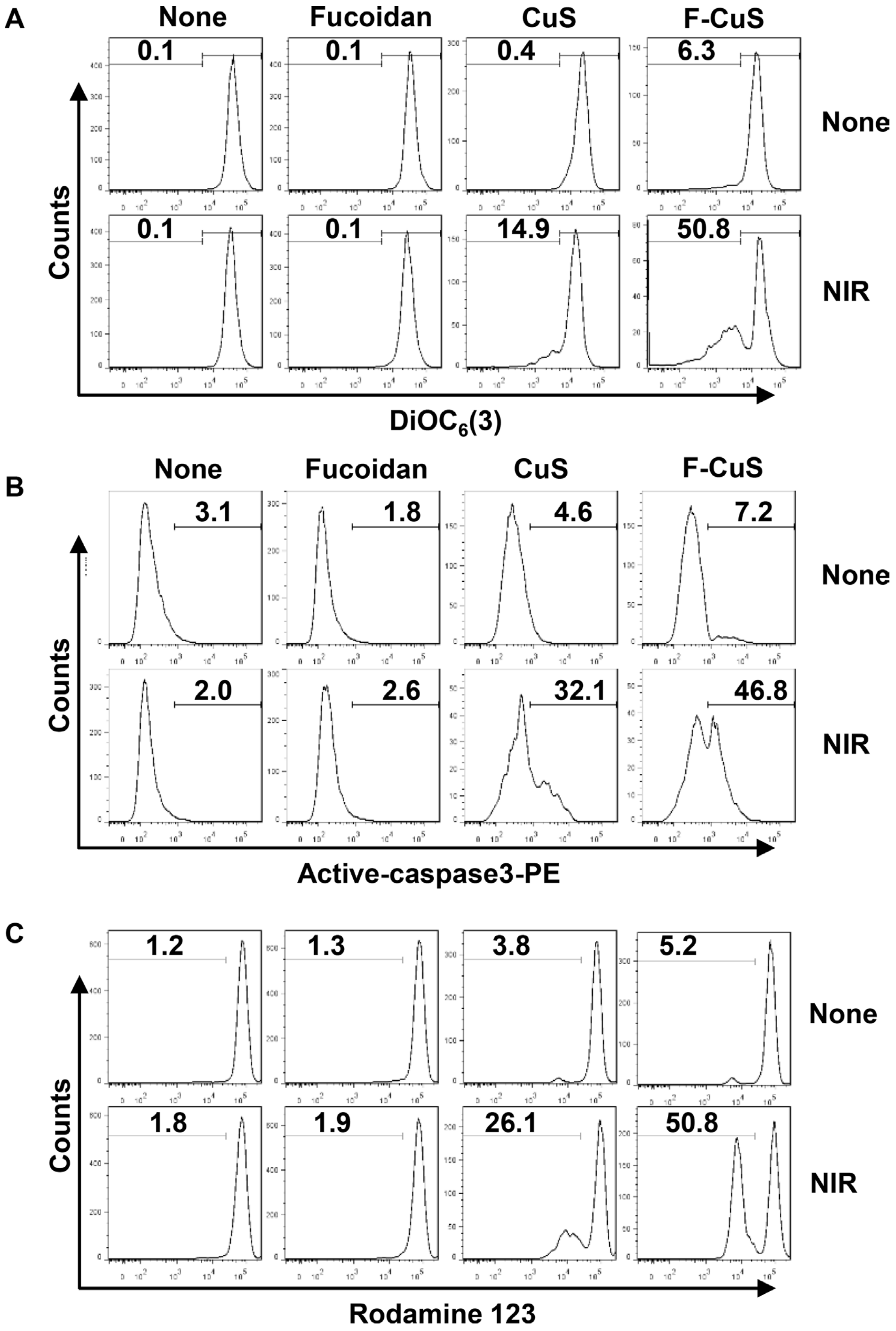

Supplementary Figure 9: Mitochondria permeability and activation of caspase 3 by F-CuS. HeLa cells (2×10^5) were incubated with fucoidan, CuS and F-CuS-FITC and irradiated with 808 nm laser for 5 min. (**A**) Mitochondria permeability was measured by $DiOC^6(3)$ reduction on a flow cytometry. (**B**) Activation of caspase 3 was analyzed by flow cytometry. (**C**) The mictochondrial membrane potential were measured by rodamine 123 staining. Data are representative of analyses of 6 independent samples (2 samples per experiment, 3 independent experiments).

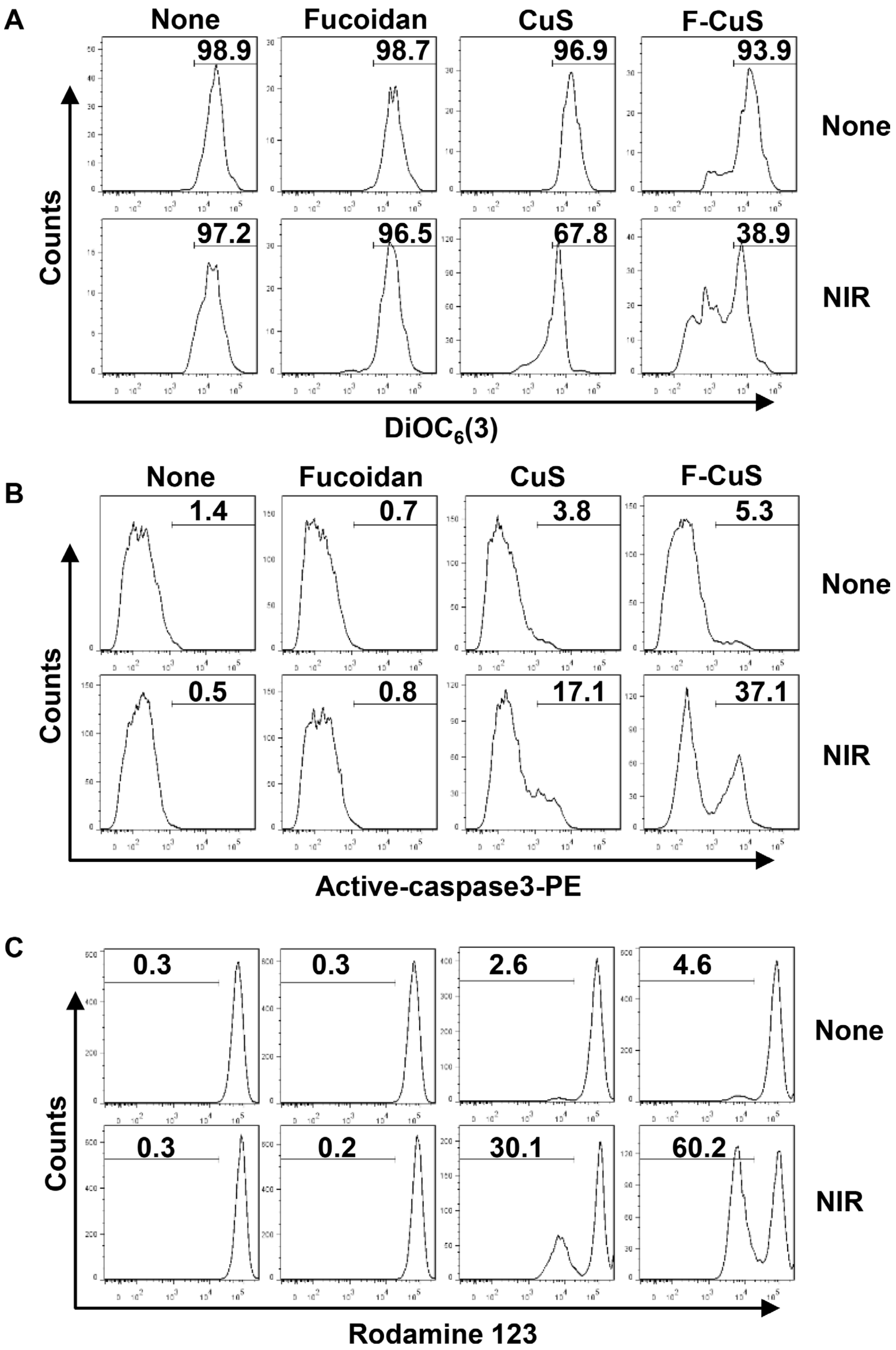

Supplementary Figure 10: Mitochondria permeability and activation of caspase 3 by F-CuS. A549 cells (2×10^5) were incubated with fucoidan, CuS and F-CuS-FITC and irradiated with 808 nm laser for 5 min. (**A**) Mitochondria permeability was measured by DiOC^6(3) reduction on a flow cytometry. (**B**) Activation of caspase 3 was analyzed by flow cytometry. (**C**) The mictochondrial membrane potential were measured by rodamine 123 staining. Data are representative of analyses of 6 independent samples (2 samples per experiment, 3 independent experiments).

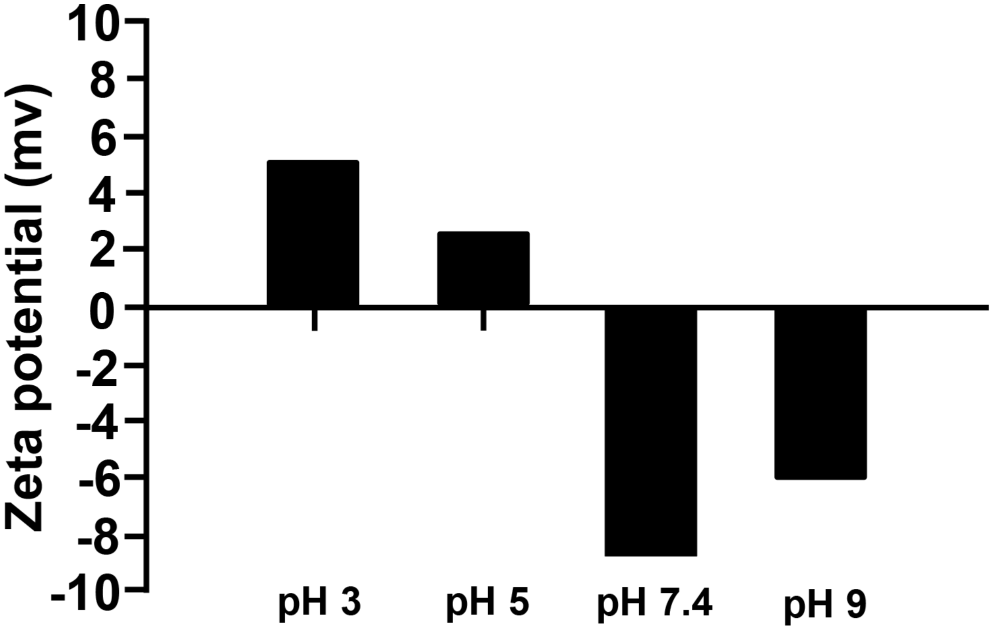

Supplementary Figure 11: Release feature of fucoidan from NPs. Zeta potential value of F-CuS changed from -8.23 (at pH 7.4) to 5.05 (at pH 3), 2.64 (at pH 5), and -5.92 (at pH 9) after 2 h exposure.

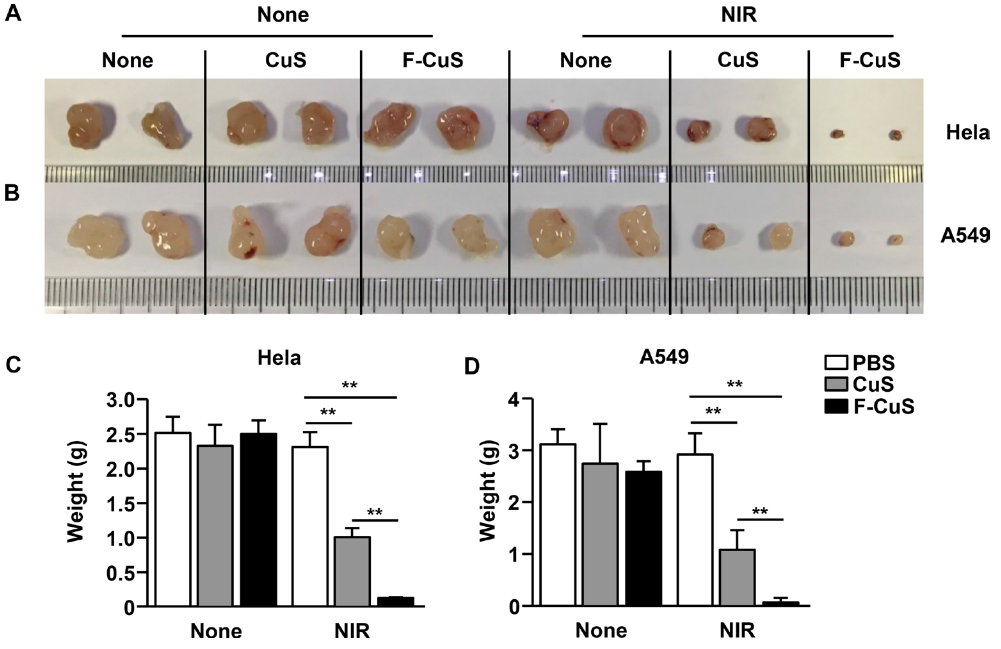

Supplementary Figure 12: Therapeutic effect of F-CuS with laser irradiation against HeLa and A549 tumor. Nude mice were injected s.c. with 5×10^6 HeLa cells and 5×10^6 A549 cells, respectively. Once tumors were measured ~5.0 mm after 14 days, the mice were treated i.t. with 40 µg/kg fucoidan, 10 mg/kg CuS and 2.5 mg/kg F-CuS. Two hours after treatment, the mice were irradiated by 808 nm laser at 2 W/cm^2 for 5 min. (**A**) HeLa and (**B**) A549 tumor mass were shown on day 35 of tumor cell injection. (**C**) Hela and (**D**) A549 tumor weight were shown on day 35. Data are representative of analyses of 6 independent samples (2 mice per experiment, total 3 independent experiments).

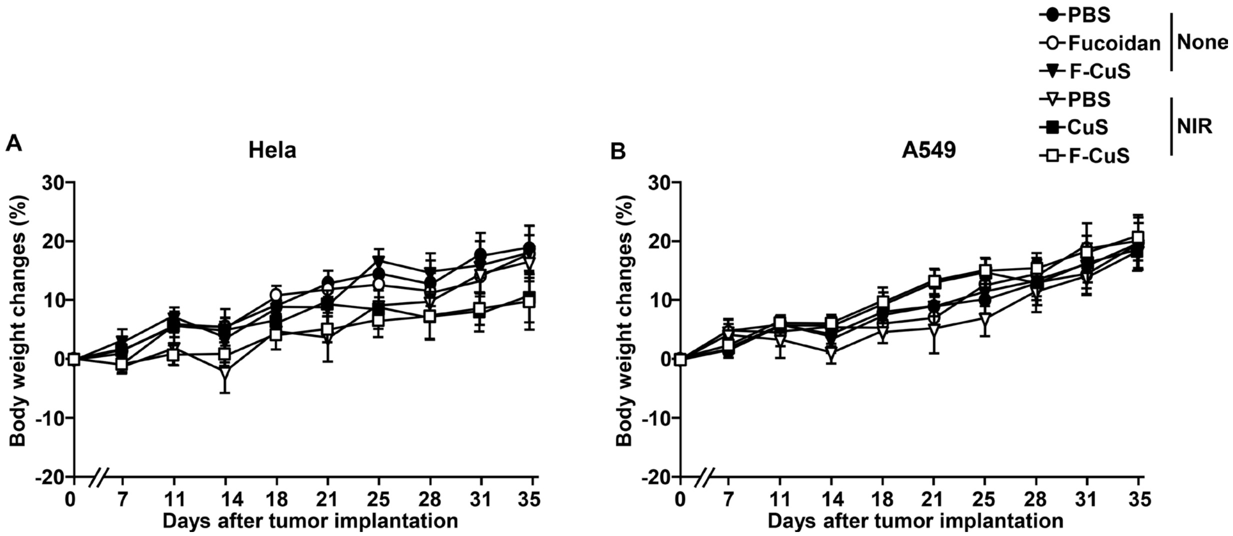

Supplementary Figure 13: Changes of body weight during treatment of HeLa and A549 tumor by F-CuS. Nude mice were injected s.c. with 5 × 10^6 HeLa cells and 5 × 10^6 A549 cells, respectively. Once tumors were measured ~5.0 mm after 14 days, the mice were treated i.t. with 40 µg/kg fucoidan, 10 mg/kg CuS and 2.5 mg/kg F-CuS. Two hours after treatment, the mice were irradiated by 808 nm laser at 2 W/cm^2 for 5 min. The body weight of (**A**) HeLa and (**B**) A549 tumor implanted mice were measured during treatment of the tumor. Data are a average of analyses of 6 independent samples (2 mice per experiment, total 3 independent experiments).

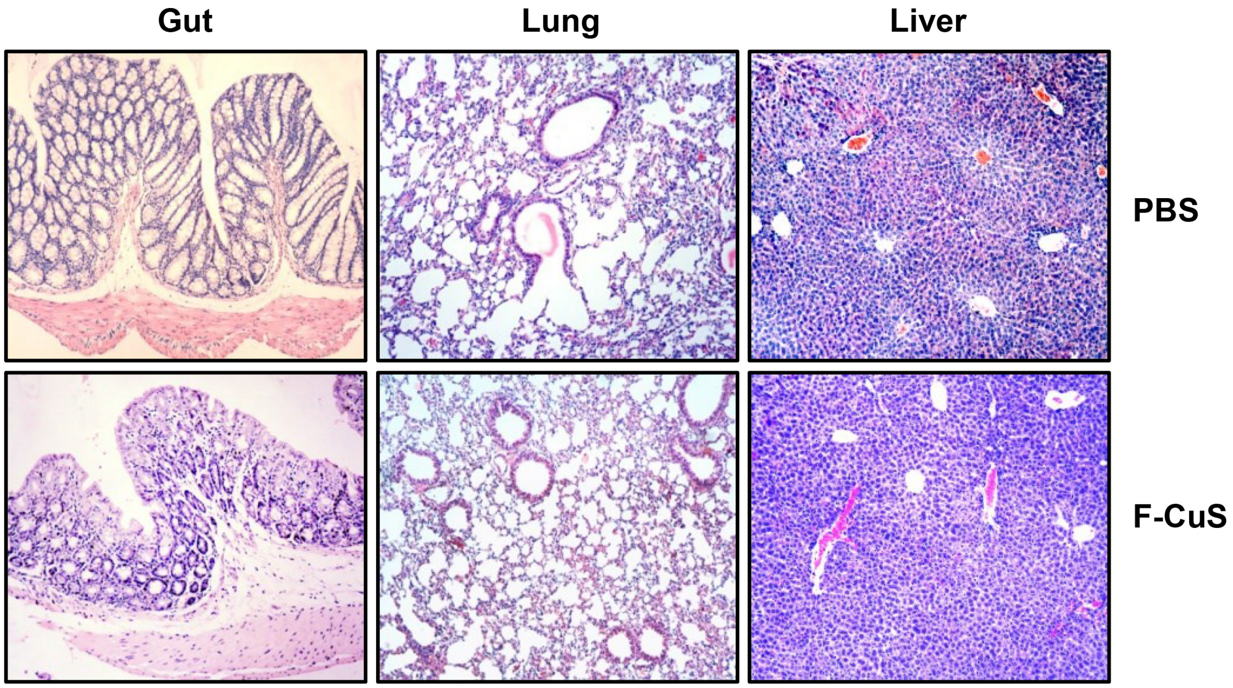

Supplementary Figure 14: Histological analysis of peripheral tissue damage. HeLa tumor-bearing nude mice were treated i.t. with F-CuS and irradiated by 808 nm laser at 2 W/cm^2 for 5 min. Twenty-one days after F-CuS treatment and laser irradiation, Gut, lung and liver were harvested and stained with haematoxylin and eosin (H&E). Data are representative of analyses of 6 independent samples (2 mice per experiment, total 3 independent experiments).

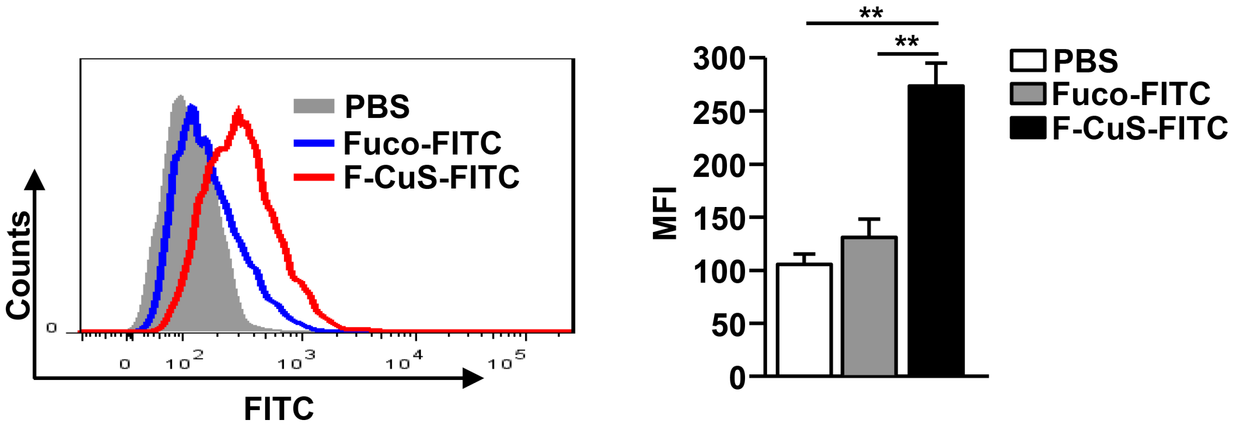

Supplementary Figure 15: Intracellular uptake of fucoidan in K562 cells by F-CuS. K562 cells (2×10^5) were incubated with fucoidan-FITC (Fuco-FITC) and F-CuS-FITC for 2 h. The intracellular uptake of fucoidan was analyzed on a flow cytometry (left panel). Mean fluorescence intensity (MFI) of FITC positive cells was shown (right panel), $^{**}p < 0.01$. Data are representative of or the average of analyses of 6 independent samples (2 samples per experiment, 3 independent experiments).

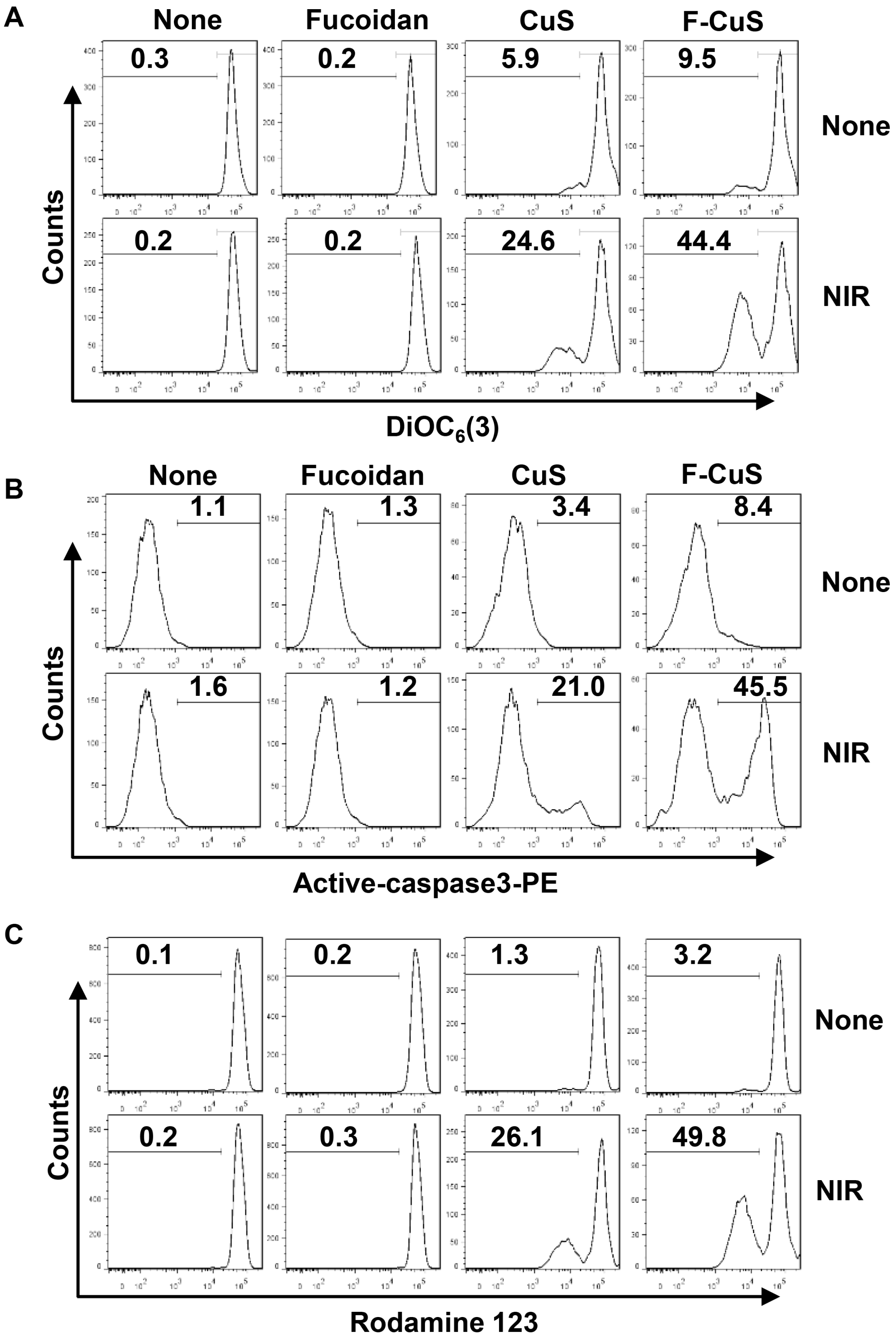

Supplementary Figure 16: Mitochondria permeability and activation of caspase 3 by F-CuS. K562 cells (2×10^5) were incubated with fucoidan, CuS and F-CuS-FITC and irradiated with 808 nm laser for 5 min. (**A**) Mitochondria permeability was measured by DiOC^6(3) reduction on a flow cytometry. (**B**) Activation of caspase 3 was analyzed by flow cytometry. (**C**) The mictochondrial membrane potential were measured by rodamine 123 staining. Data are representative of analyses of 6 independent samples (2 samples per experiment, 3 independent experiments).

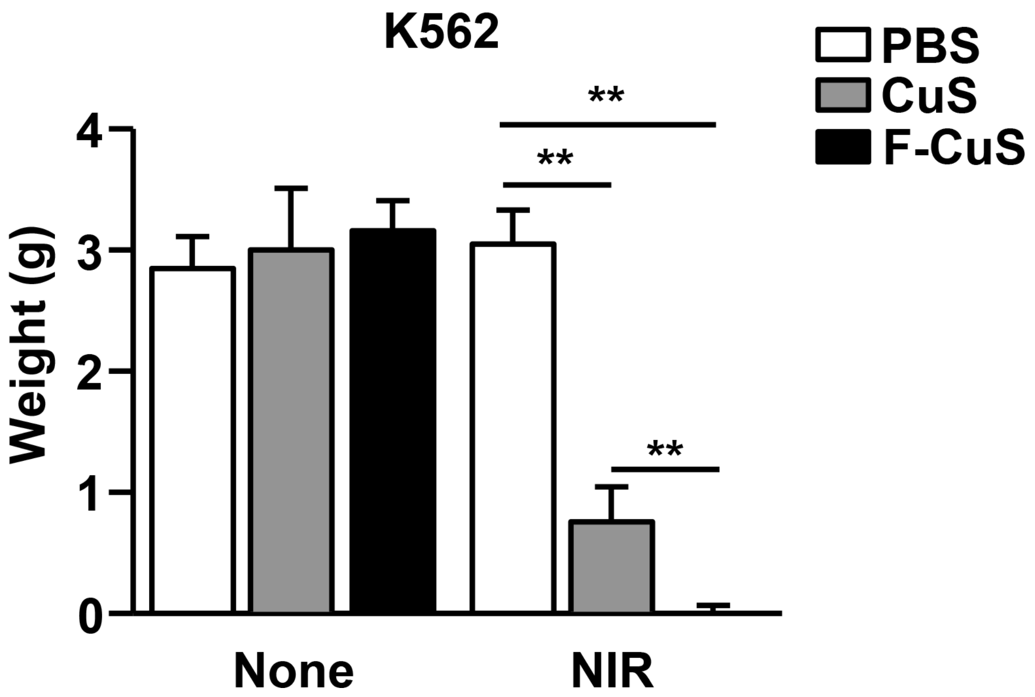

Supplementary Figure 17: Nude mice were injected s.c. with 5 × 10[6] K562 cells and treated F-CuS with or without laser irradiation as described in Figure 5C. Weight of K562 tumor mass was shown on day 35. Data are representative of analyses of 6 independent samples (2 mice per experiment, total 3 independent experiments).

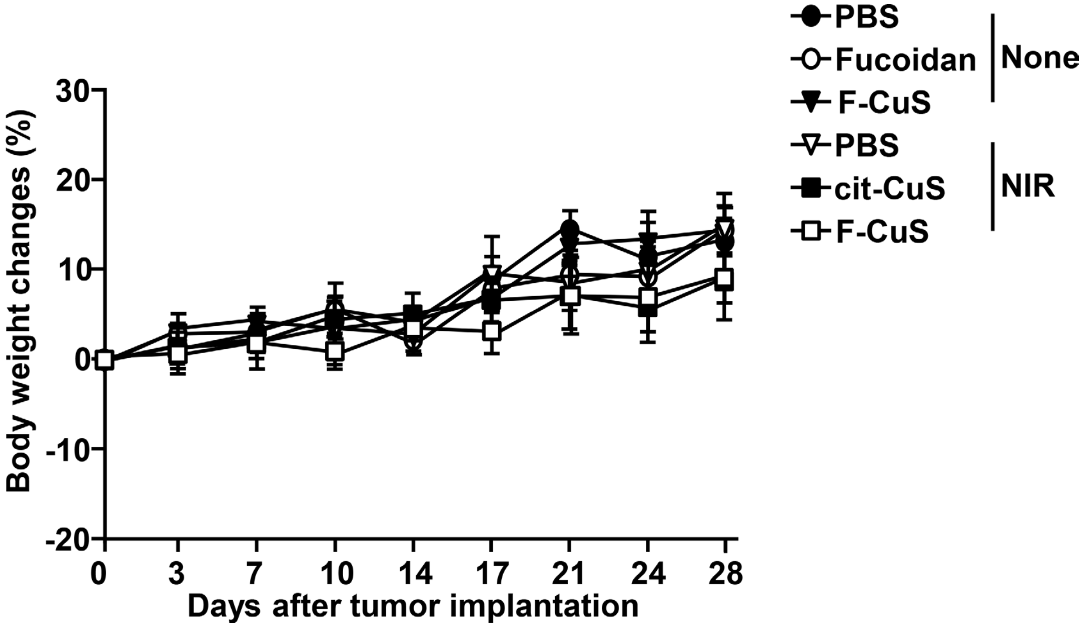

Supplementary Figure 18: Changes of body weight during treatment of K562 tumor by F-CuS. Nude mice were injected s.c. with 5 × 10[6] K562 cells. Fourteen days after tumor injection, the mice were treated i.t. with 40 μg/kg fucoidan, 10 mg/kg CuS and 2.5 mg/kg F-CuS. Two hours after treatment, the mice were irradiated by 808 nm laser at 2.5 W/cm[2] for 5 min. The body weight of the mice were monitored during treatment of the tumor. Data are a average of analyses of 6 independent samples (2 mice per experiment, total 3 independent experiments).
